# Implementing the Cognitive Orientation to Daily Occupational Performance (CO-OP) Approach with Children with Fetal Alcohol Spectrum Disorder: A Feasibility Study

**DOI:** 10.3390/children13091257

**Published:** 2026-09-16

**Authors:** Catherine Hilly, Barbara R. Lucas, Laura Miller, Peter H. Wilson, Elspeth H. Froude

**Affiliations:** 1 School of Allied Health, Australian Catholic University, Watson, ACT 2602, Australia; catherine.hilly@acu.edu.au; 2 Physiotherapy Department, Royal North Shore, St Leonards, NSW 2065, Australia; barbara.lucas@health.nsw.gov.au; 3 The John Walsh Centre for Rehabilitation Research, The University of Sydney, St Leonards, NSW 2065, Australia; 4 School of Allied Health, Australian Catholic University, Banyo, QLD 4014, Australia; laura.miller@acu.edu.au; 5 Healthy Brain and Mind Research Centre, Australian Catholic University, Melbourne, VIC 3065, Australia; peter.wilson1@acu.edu.au; 6 School of Allied Health, Australian Catholic University, North Sydney, NSW 2060, Australia

**Keywords:** CO-OP, children, fetal alcohol spectrum disorder, feasibility, motor skills, mixed methods, intervention studies

## Abstract

**Highlights:**

**What are the main findings?**

- Preliminary results on three children indicate that CO-OP shows potential to improve occupational performance and participation for children with FASD and motor coordination difficulties.
- Children with FASD can identify meaningful occupational performance and participation goals with caregiver, therapist and visual supports.

**What are the implications of the main findings?**

- More than ten CO-OP sessions (30–60 min) focusing on three goals for children with FASD may facilitate the achievement and maintenance of occupational performance and participation goals.
- Caregiver involvement supports the successful implementation of CO-OP with children with FASD.

**Abstract:**

Background/objectives: This study examined the feasibility of the Cognitive Orientation to daily Occupational Performance (CO-OP) approach for improving occupational performance, satisfaction and participation in children with Fetal Alcohol Spectrum Disorder (FASD). Methods: A mixed-methods descriptive case series explored nine feasibility domains (acceptability, practicality, implementation, expansion, limited efficacy testing, demand, recruitment rate, participation rate, and data collection). Children identified three occupational performance goals to practice across 10 weekly 30–60 min sessions. Data was collected for three children. Pre- and post-intervention scores on the Canadian Occupational Performance Measure (COPM), Perceived Quality Rating Scale- Generic version (PQRS-G) and the Participation and Environment Measure for Children and Youth (PEM-CY), along with caregiver and child interviews, were analysed. Reflexive thematic analysis examined acceptability and practicality from participants’ perspectives. Results: Five children were screened, four enrolled and three female children (*n* = 3), aged 11–12 years completed post-CO-OP measures, and their results were analysed. Caregivers and children reported some improvements in goal performance and satisfaction on the COPM. CO-OP was perceived as acceptable by caregivers and children. Caregivers valued child-chosen goals, recognised the importance of practice for progress and retention, and noted that additional support to use CO-OP strategies at home would be beneficial. Conclusions: Findings provide preliminary support for CO-OP in enhancing occupational performance and participation for children with FASD. Evaluation with a larger sample is needed to further test feasibility and determine the approach’s efficacy.

## 1. Introduction

Fetal Alcohol Spectrum Disorder (FASD) is a neurodevelopmental disorder that is defined by a spectrum of brain injuries, birth defects and developmental disabilities caused by prenatal alcohol exposure (PAE) [1]. In Australia, the estimated prevalence rate in the general population is 3.64% (95% confidence interval, 2.91%, 4.41%) and is comparable to other high-income countries, including the USA and Canada [2]. Children with FASD experience lifelong, mild-to-severe impairments in neurocognitive, behavioural, social, academic, language and motor functioning [1]. These children encounter participation restrictions at home, school and in the community caused by neurological impairments impacting daily living [3]. Motor coordination difficulties are strongly associated with FASD [4] and are included in the Australian diagnostic criteria [5]. A recent systematic review and meta-analysis found a paucity of motor interventions targeting participation outcomes for children with FASD [6].

CO-OP [7] is one intervention not previously reported in the literature for children with FASD that may support activity and participation outcomes. CO-OP has proven effective for children with other neurodevelopmental disorders that impact motor performance, such as developmental coordination disorder (DCD), attention deficit hyperactivity disorder (ADHD) and acquired brain injury [8,9,10,11]. CO-OP is a client-centred approach that combines motor learning with a global problem-solving strategy (Goal-Plan-Do-Check) to perform and achieve self-chosen goals [7]. Typically, three goals are focused on during each CO-OP session to enable children to apply the metacognitive strategy and generalise and transfer this to other occupations [7]. Learning occurs when individuals are guided to discover a strategy (plan) to resolve a performance breakdown, practice their plan, check its effectiveness and revise as needed [7]. Home practice outside of sessions supports the learning. CO-OP has been used across the lifespan for children aged four years old to older adults [8]. Studies have shown that children over the age of six years can identify their own goals for CO-OP [7]. This study aimed to examine (i) the feasibility of CO-OP using Bowen’s framework [12] and (ii) early changes in occupational performance and participation in children aged 6–12 years with FASD. Children aged 6–12 years were selected as they were likely to be able to set their own goals for CO-OP with caregiver support.

## 2. Materials and Methods

A mixed-methods descriptive case series [13] was used to explore six feasibility criteria recommended by Bowen et al. [12] (acceptability, practicality, implementation, expansion, limited efficacy, and demand). The feasibility of the study design (recruitment rate, participation rate and data collection methods) was also examined to ensure the study was practical to implement and reduce threats to the validity of the outcomes and inform future studies [14]. Feasibility was examined by converging qualitative and quantitative insights to determine whether CO-OP is a feasible intervention approach for children with FASD [12,13,14]. Qualitative data (child and caregiver interviews) explored elements of feasibility (acceptability, implementation, practicality, expansion and limited efficacy) using reflexive thematic analysis [15]. Quantitative data examined limited efficacy outcome measures pre–post intervention and at follow-up. This study was approved by Australian Catholic University human research ethics committee (Ethics ID 2022-2708H). Caregivers provided informed, written consent and children provided informed written and verbal agreement. The design and reporting of this case series followed the Joanna Briggs Institute methodological quality of case series studies guidelines [16]. This study was informed by a research advisory group (RAG) consisting of parents and caregivers of children with FASD and an adult with FASD. This RAG provided insights into the participation needs of people with FASD and identified that more participation-focused interventions would support individuals with FASD and their families.

### 2.1. Positionality Statement

The first author (CH) is a research doctoral student and occupational therapist experienced in working with children with FASD and their families. BL is a Specialist Paediatric Physiotherapist experienced in working with children with FASD and their families who holds a PhD. PW is a Professor in Developmental Psychology and an international leader in research on the motor and cognitive abilities of children with DCD. EF and LM are Professors in Occupational Therapy, experienced CO-OP therapists and certified CO-OP instructors. We recognise that our perspectives are shaped by our research and clinical experiences and have approached this study with reflexivity regarding how these may influence the interpretation and presentation of findings.

### 2.2. Participants

Inclusion criteria. Children aged 6–12 years who lived in New South Wales (NSW) and the Australian Capital Territory (ACT) were eligible to participate if they had a confirmed diagnosis of FASD made by a paediatrician, had motor coordination difficulties and with support were able to identify 3–5 goals for therapy. Confirmation of a diagnosis was reported by caregivers as being made by a paediatrician using the Australian Guide to the Diagnosis of Fetal Alcohol Spectrum Disorder [17].

Participants were recruited through FASD diagnostic services and the Australian National Organisation of Fetal Alcohol Spectrum Disorder (NOFASD). NOFASD promoted the study through public social media posts and advised their members of the study. A FASD diagnostic service shared recruitment fliers with children diagnosed with FASD with motor impairment. Recruitment was open from August 2023 to June 2025, and in this time, caregiver participants expressed an interest online through REDCap (version 17.1.3-2026), an electronic data capture tool hosted at Australian Catholic University [18,19]. All participating children were screened prior to commencing CO-OP by CH and met the inclusion criteria. Motor difficulties were identified by caregivers completing the Developmental Coordination Disorder Questionnaire (DCD-Q) [20] and children completing the short form of the Bruininks Oseretsky Test of Motor Proficiency, second edition (BOT2-SF) [21], administered by CH. Participants with scores on the DCD-Q qualifying as “probably DCD” and a total BOT2-SF at or below the 16th percentile were included.

Exclusion criteria. Children without a confirmed diagnosis of FASD made by a paediatrician, unable to participate in goal setting, living outside of the geographical area (ACT and NSW) or with no movement difficulty identified were ineligible.

### 2.3. Procedures

Children identified a maximum of five goals for therapy using the COPM and the Perceived Efficacy and Goal Setting System [22] with CH and caregiver support. Three goals were prioritised by the children for therapy. The remaining two were noted as unpracticed goals. Goals focused on performing meaningful daily occupations to improve functional participation and independence. A picture-based tool, The Perceived Efficacy Goal Setting System 2nd Ed. [22] was used to generate discussion about possible goals. Visual analogue scales of a ladder and faces were used to assist children to rate their performance and satisfaction with their goal performance on the COPM [23]. This approach was adopted from a similar study used by Araujo et al. [24]. Goals identified by children included home occupations (keeping room tidy, spreading with a knife, retrieving/putting away items from/to the fridge, cracking eggs to feed the dog), self-care occupations (tying shoelaces, washing own hair), school occupations (writing on the lines, reading long words) and leisure occupations (riding a bike without training wheels, knitting).

CO-OP sessions were delivered by a certified CO-OP therapist (CH). EF, an experienced CO-OP instructor and therapist, provided coaching based on reviewing video sessions of sessions 1–3 of each participant. Intervention consisted of 10 × 30–60 min CO-OP sessions, in addition to an initial screening and goal setting session, provided weekly at the child’s house after school. Home environments were selected for family convenience, and to enable children to practice their goals in their lived environments. Prior to the first intervention session, children and caregivers were emailed an information booklet [25] about the CO-OP approach. CO-OP was described to children and caregivers in the first session by the therapist. Where possible, sessions were video recorded following consent to evaluate treatment fidelity and to analyse the participant’s involvement in CO-OP and the child’s goal performance. Field notes were documented after each session to record participants’ responses to CO-OP, goal progression and reflection on implementation.

The global strategy of ‘Goal-Plan-Do-Check’ [7] was introduced during the first session and reinforced at the subsequent nine weekly sessions. The CO-OP sessions focused on children learning to problem solve, discover strategies and develop plans to overcome performance challenges with the same three goals. Home practice supported consolidation of the strategies and plans developed in the sessions. Each session began with a review of home practice and practiced strategies to problem-solve barriers to perform the goals. Guided discovery, allowing child participants to problem solve their own performance difficulties instead of direct instruction, was used to coach the children to develop their plans to achieve their goals, explore domain specific strategies, evaluate their progress and identify their performance breakdowns [7].

### 2.4. Data Collection

Feasibility criteria were primarily evaluated qualitatively with quantitative measures to support data interpretation. Criteria to evaluate feasibility were established a priori (Supplement File S1). The following section describes the data collected. Data were collected at three pre-planned timepoints (T1, T2, and T3) (Table 1) of the study.

Acceptability was assessed using the Parents as Partners in Intervention Satisfaction Questionnaire (PAPI III) [26] and by asking children and caregivers about their experiences participating in CO-OP using semi-structed interviews.

#### 2.4.1. Parents as Partners in Intervention Satisfaction Questionnaire (PAPI III)

The PAPI III [26] is a criterion-referenced tool and has moderate internal coefficients and criterion validity when compared to the COPM. It uses a five-point Likert scale asking caregivers about their satisfaction with an intervention. Higher scores on the PAPI III indicate higher satisfaction and lower scores indicate low satisfaction.

#### 2.4.2. Parent and Child Interviews

Interviews (Supplement File S2) were conducted by CH, who implemented CO-OP. Children and caregivers were invited to be interviewed by another independent member of the research team; however, they preferred to be interviewed by the researcher whom they had established rapport with.

#### 2.4.3. Field Notes, Reflective Journal, Session Record, Home Practice Log

Child and caregiver data and PAPI III scores were analysed with field notes and video data to determine acceptability and enjoyment during sessions. Practicality (ease of use) (the extent, likelihood and manner in which CO-OP can be delivered when resources, time, or commitment are constrained in some way) was evaluated using field notes and a reflective journal of session records, and home practice log discussions with children and caregivers. A home practice log was developed and provided to participants for the study with the intention of children and caregivers recording the practice of each goal and strategy use weekly (Supplement File S3). Demand and study design were assessed using recruitment, retention and participation rates, as well as the completion of all measures.

Implementation (the extent to which CO-OP was implemented as planned) was evaluated using the CO-OP Fidelity Checklist [27] and evaluating children’s engagement in video recorded sessions with the Pediatric Rehabilitation Intervention Measure of Engagement—Observation (PRIME-O) [28].

#### 2.4.4. CO-OP Fidelity Checklist

An experienced CO-OP therapist and certified instructor evaluated the fidelity of the goal setting session and two additional intervention sessions. She was not blind to the participant’s intervention status. The CO-OP Fidelity Checklist was used to score adherence to the approach [27,29]. The CO-OP Fidelity Checklist [27] involves evaluating intervention fidelity in three parts. Part A.1 evaluates fidelity across sessions against six criteria. Part A.2 evaluates fidelity within sessions against 11 criteria. Part B evaluates general CO-OP fidelity against 9 criteria. Each criterion is rated as No, Yes (1–5) or NA/NO (not available/not observed). To provide more nuanced feedback items were scored on a Likert scale from 1 (does not do well) to 5 (does very well) compared to the yes, no, N/A responses in the checklist. Higher scores indicated good CO-OP intervention quality. Fidelity across sessions, within sessions and general CO-OP fidelity was evaluated. Fidelity scores for each section (i.e., fidelity across sessions, within sessions and general CO-OP fidelity) were calculated by adding the total ratings (1–5), dividing by the maximum possible score (excluding N/A or N/O ratings) and multiplying by 100 to convert to percentages.

#### 2.4.5. Pediatric Rehabilitation Intervention Measure of Engagement—Observation (PRIME-O)

The PRIME-O [28] measures child and service provider affective, cognitive and behavioural involvement in intervention sessions and has excellent interrater reliability and takes 10–15 min to complete 10 ratings [30]. It measures levels of engagement on a Likert scale from 0 (not at all) to 4 (to a great extent) within client, service provider and client-provider items. A total score of 40 is possible, with highest scores indicating full engagement. PRIME-O measures were completed by an experienced paediatric physiotherapist (BL) who was a member of the research team with available data across four sessions per participant (Table 2). BL was not blind to the participant’s intervention status.

#### 2.4.6. Demographic Data

Demographic data was collected through a caregiver questionnaire (Supplement File S4) and an evaluation of the child’s adaptive behaviour measured by caregiver ratings on the Vineland Adaptive Behavior Scale Third Edition domain level form (VABS-3) [31]. The VABS-3 is a caregiver-rated questionnaire that assesses communication, daily living skills and socialisation. This information is then used to determine an overall adaptive functioning measure called the adaptive behaviour composite. Higher scores indicated higher adaptive behaviour than lower scores.

#### 2.4.7. Outcome Measures

Expansion of CO-OP to an unpublished population of children with FASD and limited efficacy (testing immediate rather than final outcomes, with shorter follow-up and limited statistical power) were evaluated using quantitative measures: the COPM [23], the PQRS-G (generic version) [32] and the PEM-CY [33]. Table 1 provides a timeline of data collected from baseline to end of study outcome measures at 3- or 6-month follow-up post final session (S10). Data was collected at three timepoints: baseline (T1), S10 (T2), and 3- or 6-month follow-up (T3). Note that planned follow-up at 3 months could not be completed for two of the three families due to difficulty reconnecting. T3 for these two families occurred at 6 months.

##### Canadian Occupational Performance Measure (COPM)

The COPM measures change in clients’ perceived performance and satisfaction with meaningful goals [23]. It has strong test–retest reliability varying from 0.84 to 0.92 [34]. A visual analogue scale is used to facilitate performance and satisfaction ratings from 1 (not performing well and not satisfied) to 10 (performing very well and very satisfied) [23]. A two-point change is considered clinically meaningful [23]. The COPM was rated by children and caregivers for their perceived satisfaction with and performance of each goal at three timepoints (Table 1). It was administered by the therapist providing CO-OP (CH) who was not blinded to the participant’s intervention status to reflect clinical practice and build rapport with participants. Child and caregiver COPM ratings were reported alongside PQRS-G data.

##### The Perceived Quality Rating Scale—Generic (PQRS-G)

The PQRS-G is an observational, video-based tool that measures child participants’ observed performance on their self-chosen goals [32]. Quality performance is rated on a 10-point Likert scale ranging from 1 (skill is not done at all) to 10 (skill is performed very well). Each chosen activity is performed by participants without verbal or physical guidance in a non-standardised environment. It has moderate interclass correlations (ICC) varying from 0.71 to 0.77 and substantial test–retest reliability for all categories of raters and large effect sizes (greater than 1) [32]. Smallest real difference scores for children ranged from 2.13 to 2.91, thus a change in score of 3 is required for 95% confidence certainty of change. The PQRS-G evaluated each child’s video recorded goal performance at three timepoints (Table 1). It was rated by an experienced CO-OP therapist and instructor blinded to the participant’s intervention status.

##### Participation and Environment Measure for Children and Youth (PEM-CY)

The PEM-CY [33] is a criterion-referenced questionnaire completed by caregivers about their child’s participation (aged 5 to 17 years). It measures frequency, type of activities and level of involvement and influence of the environment on participation in 25 activities across home, school, and the community [33]. Frequency of engagement in each setting (home, school and community) is measured on an eight-point Likert scale (0 = never to 7 = daily). Highest scores represent greatest frequency across activities [32]. Level of involvement was rated on a five-point Likert scale (1 = minimally involved to 5 = very involved) and items were averaged [33]. Caregivers completed all three settings (home, school and community) at three timepoints (Table 2). Averages of frequency of participation and involvement in all three settings were calculated by summing the total ratings in each setting and dividing these by the number of ratings made. PEM-CY data was collected from caregivers electronically in REDCap [18,19]. The PEM-CY [33] was used to explore any potential changes in participation frequency or involvement following CO-OP.

### 2.5. Data Analysis

Immediate outcomes (limited efficacy) and the potential to apply CO-OP to children with FASD (expansion) were examined by evaluating occupational performance and participation of child-identified goals at pre-, post- and follow-up assessments by comparing COPM, PQRS-G and PEM-CY scores and interviewing caregivers after children completed CO-OP about their child’s participation in daily activities. COPM, PQRS-G, and participation data were extracted into Excel spreadsheets (Microsoft Corporation) and cross-checked for consistency. Descriptive statistics (such as percentages) were calculated in Excel [35] to evaluate feasibility criteria (Table 1). Further statistical testing was not undertaken due to the sample size and feasibility study aims.

Interviews were transcribed verbatim and analysed using the six phases of reflexive thematic analysis described by Braun and Clarke [15] and acknowledging CH’s active role in the analysis. The transcripts were read several times and coded with semantic and latent approaches. Deductive coding was applied after inductive coding. Coding initially represented CH’s interpretation and meaning across the dataset before applying Bowen’s feasibility criteria to gain insights into acceptability, practicality, implementation and limited efficacy [12] (Supplement File S5). Codes were assembled into six initial candidates based on themes and later refined into six themes with central organising concepts around shared meaning (Learning and accepting CO-OP; Practice influences outcomes; Child engagement in CO-OP; Family involvement in CO-OP; Goals; Working towards independence and participation) [15]. Trustworthiness was maintained by keeping a reflective journal and a coding Excel spreadsheet [35] to document the analysis and aid reflection. CH gained feedback from the research team to strengthen her reflexivity around codes and candidate themes [15,36]. Engagement with participants over a long period of time was maintained as CH provided the CO-OP therapy and interviewed participants at T2 and T3 [15,36]. Convergent analysis of quantitative and qualitative findings [13] was integrated to provide context to the variability in goal achievement and experiences of children and their caregivers. Both qualitative and quantitative data were collected and analysed simultaneously. Themes from qualitative data were consistent with quantitative data, including PAPI III scores and PRME-O scores. Variations in COPM data between children and caregiver ratings and PQR-S-G scores were presented as raw data and changes in scores at T2 and T3. Themes are reported under each of Bowen’s feasibility criteria [12].

The first author (CH) delivered the CO-OP intervention, administered the COPM to children and caregivers, conducted the interviews and led the qualitative analysis. CH developed rapport and trust with participants who were offered the opportunity to be interviewed by other members of the team. It is recognised that this may create a risk of expectation and social desirability responses from participants. However, COPM scores from children and caregivers were supported by PQRS-G scores and field notes. Rich descriptions of events and direct quotations from participants were reported to enhance quality of findings [15].

## 3. Results

### 3.1. Description of Participants

**Figure 1:**
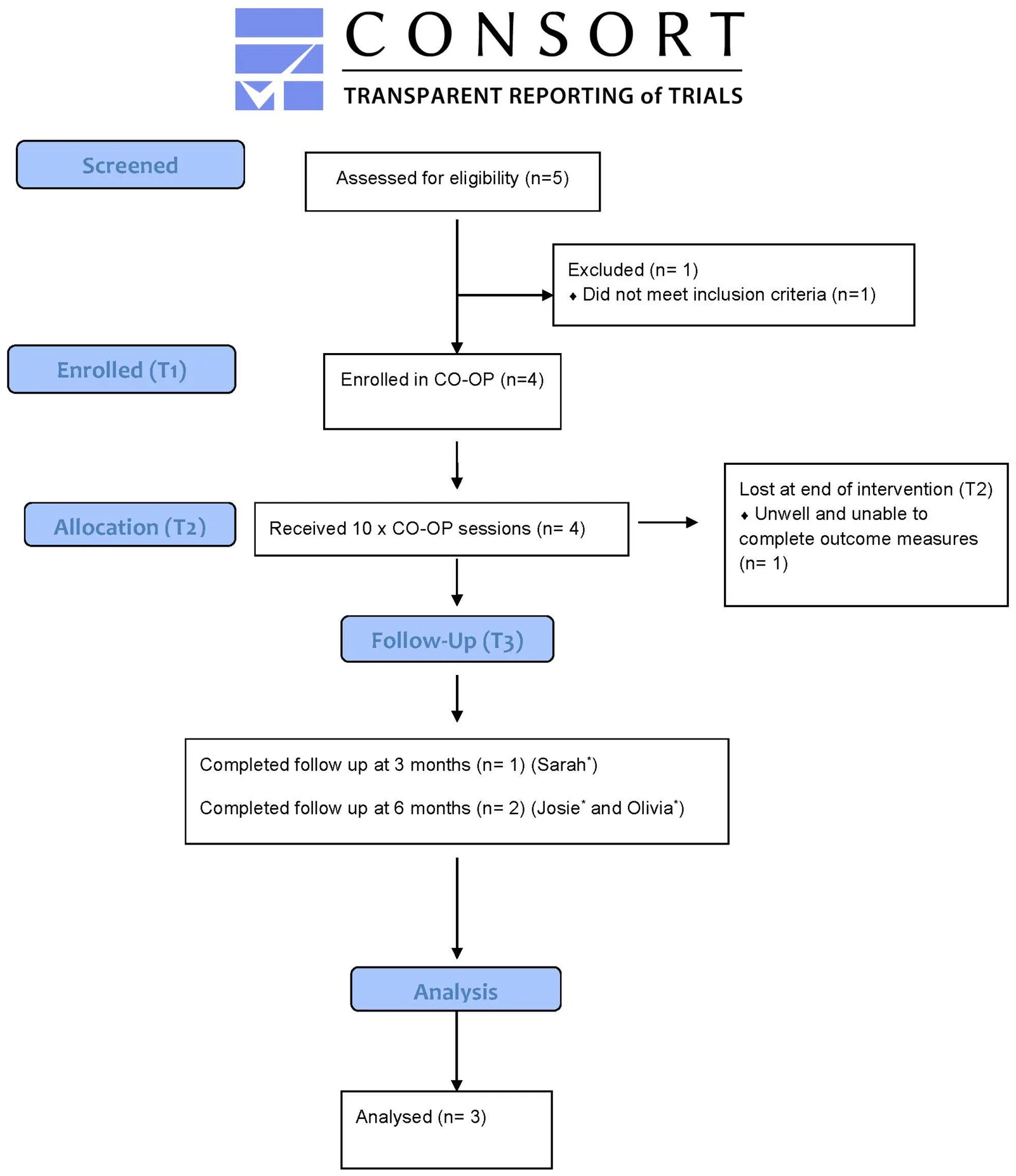
Participant flow diagram. * pseudonyms used.

There were five children screened in this study, of whom four children were determined eligible. Of the four determined eligible, all provided consent and were enrolled in this study. One screened participant, participated in all 10 CO-OP sessions and could not proceed with post-CO-OP data collection due to health and contextual factors. The data from this participant was removed from the study as it was incomplete (Figure 1).

The three children who participated completed all stages of the CO-OP process and the follow-up session. These children had a confirmed diagnosis of FASD made by a paediatrician as reported by caregivers, were identified as female and were aged between 11 and 12 years. The demographic information is provided in Table 2. Two children had additional co-occurring diagnoses of autism. Pseudonyms were used to deidentify child and caregiver participants when analysing the results. Of the three participants, two could not be contacted for follow-up until six months post-CO-OP. This is reflected in the participant flow diagram (Figure 1).

The results of this study are presented under Bowen’s feasibility criteria [12]: acceptability, practicality (ease of use), implementation, expansion, limited efficacy and demand. The feasibility of the study design, including measuring recruitment rate, participation rate and data collection methods, was also evaluated. Table 3 presents a summary of the feasibility criteria findings. With three participants, an 80% threshold requires all three participants to endorse each criterion.

### 3.2. Acceptability

All caregivers reported acceptance of and satisfaction with CO-OP. They identified that CO-OP was different from other approaches that they had used with their children. For example, Greg reported, “*It’s pretty hard to compare it to something else. It was a bit different.*” Helen reported initial frustrations with implementing CO-OP. She was frustrated with the process and wanted to directly tell her daughter what to do, rather than let her practice the problem-solving strategies. She reported:

*I guess for me, it was a little bit frustrating with the plan part [of CO-OP] letting her try to do the planning part when it wasn’t working… so from my perspective it was difficult ‘cause I could see and it wasn’t working…and result in her just getting frustrated and, so um, like a couple of times it was like it’s not working so I’m going to show you the steps to do it even though I know that’s not technically [CO-OP]* (Helen).

However, during the intervention period, Helen began to step back and allow her daughter to have a go and while using guided discovery. She described the following:

*we were doing it more with the hair washing, it was just like wetting the hair, and…. giving her general directions but letting her work it out* (Helen).

Both Greg and Kate valued the CO-OP protocol and felt that it supported their children’s learning and participation. Greg appreciated the stepped-out approach of CO-OP that allowed his daughter to anticipate what was required of her, and said, “*I think that any sort of protocol that’s stepped out like that is effective to use with her because of what’s coming up.*” Kate described the CO-OP approach as “*targeted*”, describing the repetition of practicing the same three goals every session to be beneficial to her daughter. She stated, “*she knew what, like every time you came, it was a Monday. She says to me, is the egg lady [occupational therapist] coming today?*” and “*you just gotta have that patience and the repeatability of the order that you do things*” (Kate). Kate found the approach helpful for her to support her daughter, even though she recognised that her daughter could not implement the metacognitive strategy (Goal, Plan, Do, Check) independently by the end of the study. She said:

*But for me to have a way of getting her to do something, it’s good for me to talk to her about something [using Goal, Plan, Do, Check] and then do it in that fashion* (Kate).

All caregivers appreciated their children identifying their own occupational performance goals, which is a key feature of CO-OP [7] and recognised that these helped their children’s motivation and involvement. For example, Helen said:

*I think its [choosing her own goals] good because, they’re things that she finds frustrating. Her being able to choose things and then she is more likely to practice it and it’s gonna have a bigger impact on her life because they are things that frustrate her*.

Greg recognised his daughter’s motivation when she chose her own goals and commented, “*especially when I can see goals that she’s set for herself because she obviously has her own agenda to achieve them*.” Kate also recognised the influence of her daughter’s choice on her motivation and reflected:

*you’d think that she’d be much more inclined to engage with it if it’s on her terms, which we know that’s how she operates. Everything has to be on her terms. It always went back to her chosen one was what she wanted to do* (Kate).

The scores on the PAPI III satisfaction questionnaire [26] confirmed caregivers’ satisfaction with CO-OP. Of the three caregivers, two reported strong satisfaction with CO-OP and one (33%) rated slight satisfaction.

All three caregivers reported that they would like more than the ten CO-OP sessions to reinforce their children’s learning and continue to develop their goals. Helen commented, “*I think more sessions would probably…we would have got a little bit further.*” Greg stated, “*you know, people like [Olivia] it’s an ongoing thing. I don’t think there could ever be enough sessions*.” Kate reflected on her daughter’s bike riding goal:

“*They only happened towards the end [bike riding]. So, if we had all the sessions again, just imagine, she’d be totally in*” (Kate).

The children’s responses were mixed. Josie found doing CO-OP “*stressful*” because she had to evaluate her performance and make decisions, which she had not previously experienced. She also reported that there were “*too many goals*” to address. In contrast, Olivia enjoyed doing CO-OP and “*learning the approach*” and having regular time with the CO-OP therapist. She did not identify anything that she disliked. Sarah only enjoyed CO-OP “*a little bit*” because she “*liked to learn new things*.” All three children reported that they would recommend CO-OP to other children with FASD because “*they get to practice doing things*,” (Joise), “*helps kids get into a routine*” (Olivia) and “*helps them learn*” (Sarah).

### 3.3. Practicality (Ease of Use)

All 10 sessions were implemented for all participants. Factors that may have supported this were that the CO-OP therapist travelled to the participant’s homes and scheduled appointments flexibly with participants after school hours. All children practiced using the cognitive strategies with the therapist during sessions and practiced at least one of their goals outside of the CO-OP sessions. Helen and Greg acknowledged family and child challenges with home practice. Helen stated, “*but again it’s that, you know having time and effort to get to a point where she’s better at developing her own plan*” and Greg reflected:

*I haven’t really used it to be honest with you… It was more of a consequence of a bit of a hectic lifestyle we’ve been living at the moment. With me working away a lot and [partner] effectively being a single mum with three kids for a lot of the year. There is just not a lot of time to reinforce it and keep going over and over it again* (Greg).

Family involvement was important to the child’s goal success. Helen and Kate were present for every session to support their child’s goal practice. Although Greg was not available for every session to support Olivia, she mostly practiced her handwriting goal independently at school and tidied her bedroom weekly with her stepmother. Kate embedded practice into her child’s daily routine:

*[Practice] we did that. We did that more with the knitting as a bedtime activity cause it’s so it’s kind of you fall asleep while you’re doing it quite easily. So, it was a good calming sort of activity to do together* (Kate).

During one CO-OP session with Sarah, her cousin was present. Her cousin, who was of a similar age, could not yet ride a bike. Kate noticed that her daughter was more motivated to ride her bike after her cousin’s visit, as she described:

*And the other thing I think that was helpful was when [cousin] said to her, she has no clue on how to ride a bike and that she wanted you to give her lessons. I think that was a clincher because [cousin]’s better at [Sarah] in virtually everything. And all of a sudden, [Sarah]’s like, I can ride a bike and [cousin]’s going, I wish I could be like you. And that was really, really critical* (Kate).

Kate recognised that, as her daughter’s main caregiver, she was best placed to support her daughter to participate in CO-OP and valued the collaboration with the occupational therapist. She stated:

*And that they really need the parents to participate there as well, because otherwise they just take over, do their own thing more, whereas I, I can keep her down, attending to it… You don’t always get the same outcomes from it, but you’re more likely to get them to attend if you’re there with them. Yeah, that’s so important to because then the therapist teaches you how to continue to do it because they’re not there every day and they’re only there for a short period of time. So, to have that knowledge and skill and to see that it works…It was really helpful for me anyway* (Kate).

By contrast, Helen identified that she needed support to implement CO-OP and goal practice with her daughter:

*I think we got to a stage where we were at where we need someone to do it with us. We’ve been doing a lot over the years. A lot of stuff just the two of us…to the point now where she is, she finds it frustrating and gets angry with me. If [therapist] had come over a few more times you would have got a little bit better at it than you are because you don’t do it anytime that I ask you to do it [talking to Josie]* (Helen).

Another factor that influenced the ease of use of CO-OP was cognitive and physical fatigue. This was only identified by Helen, who explained, “*I think that affects her ability to [participate in CO-OP], that then links in. The physical being physical tired links in with her frustration tolerance and her ability to plan and do tasks*” (Helen).

**Figure 2:**
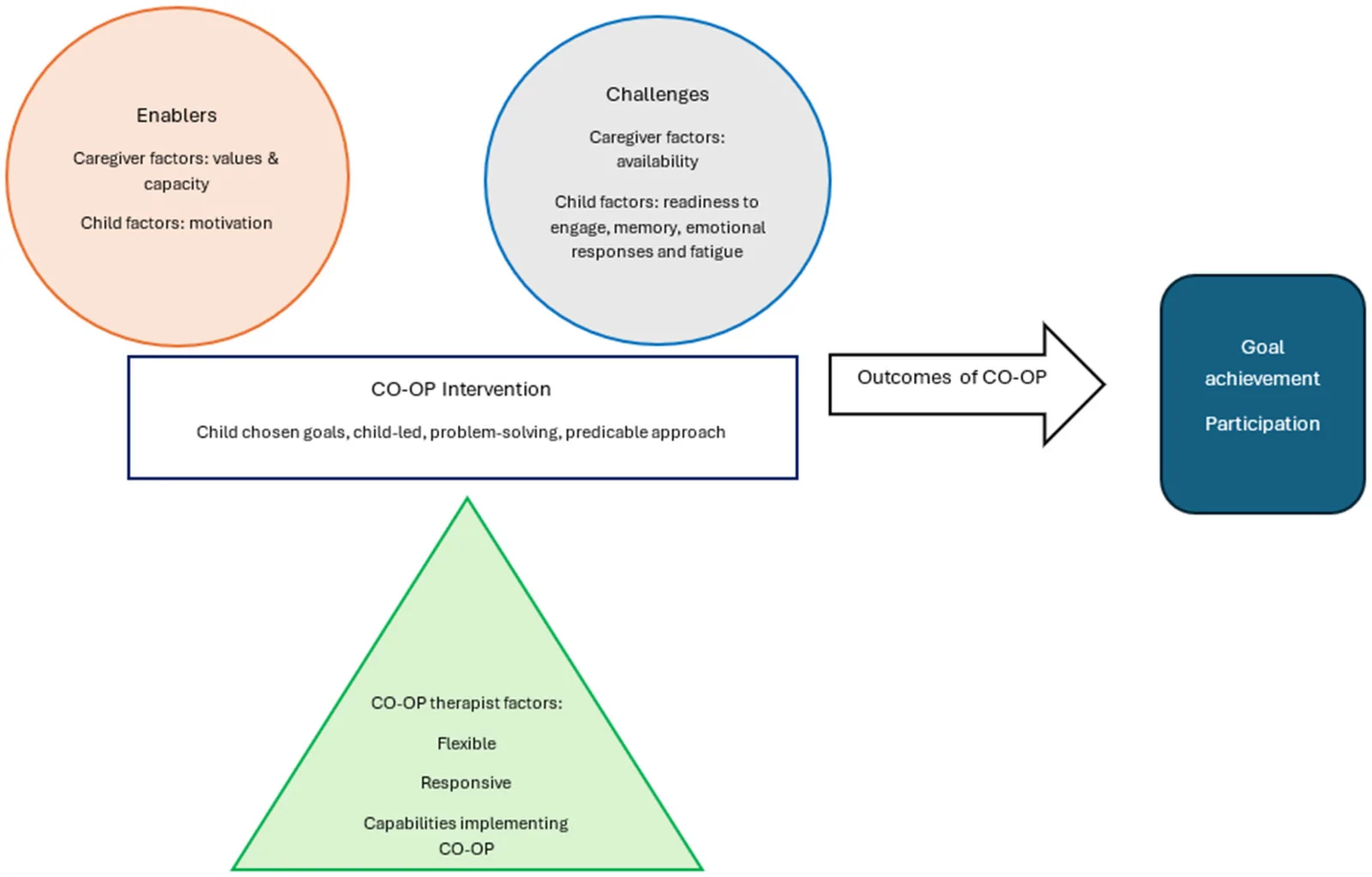
Enablers and challenges to goal achievement and participation.

Several enablers and challenges to using CO-OP to support goal practice and achievement were identified. Challenges included (i) caregiver availability to participate in CO-OP sessions, and (ii) child factors: readiness to engage, emotional responses, memory, and fatigue. Enablers included (i) caregiver values and capacity and (ii) child motivation. CO-OP therapist factors and the CO-OP protocol mediated the enablers and challenges. CO-OP therapist factors included (i) flexibility and (ii) responsiveness to the child and caregiver and (iii) capability in implementing CO-OP (Figure 2).

### 3.4. Implementation

Josie and Olivia completed goal setting in one session (S1) and outcome measures (COPM) at three timepoints (T1, T2 and T3 at 6-month follow-up). Sarah completed goal setting at S1 and S2 and outcome measures (COPM) at T1 and T2. Sarah took two sessions to identify three goals and become familiar with the occupational therapist and the CO-OP approach. Olivia chose to discontinue working on her second goal of keeping her school bag tidy at S7, and therefore this was not scored at T3 but was at T2. Caregivers also completed the COPM and the PEM-CY at all three timepoints. Caregivers (Helen and Greg) reported that the PEM-CY was repetitive, and Greg did not complete the full PEM-CY at T2 or T3. Helen and Kate completed all sections of the PEM-CY at each timepoint of the study.

A home practice log was developed for the study but was not completed the way it was intended with caregivers and children documenting home practice each week. Instead, caregivers and children found informal discussions at each session more helpful for reporting information about CO-OP home practice outside of the therapist-supported sessions.

Josie participated in 100% of sessions and these were all video recorded. She experienced difficulties engaging in the CO-OP process and found it “*stressful*” and “*frustrating*” and had difficulty remembering the metacognitive strategies. Josie stated, “*I keep forgetting [to use GPDC]. I also, forgot about that as well [re: checking]*.” Her mother described, “*it’s a pretty common FASD thing…to analyse and use other information in new situations is a really big issue with her*” (Helen). Helen also acknowledged Josie’s frustration in relation to her goal of tying her shoelaces:

*I don’t really know because she was doing really well for, and then she just had one morning where it was just not happening for her. And from then on, she seems to, and as I said if there was only one shoe. I think she would be better but it’s that frustration level that she doesn’t have a huge tolerance for getting frus, like she gets frustrated quite easily* (Helen).

Olivia’s involvement was confined to 70% of the sessions, and these were not video recorded. Her mood was judged as low during S2 and S9, and she declined to engage in guided discovery but was happy to talk to the therapist and draw. Olivia agreed to check her goal performance in session five and agreed to videoing of some goal performance (writing). At the 6-month follow-up session (T3), she did not agree to perform her goals or have the session recorded. Instead, she agreed to share a sample of her writing in a schoolbook. She reported that she had not used CO-OP since the intervention sessions ended because “*I got distracted*.” Her father reflected on Olivia’s engagement in CO-OP:

*Like everything else, it is dependent on a bunch of variables that neither you nor I really know [Olivia’s response to participating in CO-OP]. I think it just came down to the mood on the day* (Greg).

Sarah participated in all sessions yet only allowed parts of the sessions (80%) to be recorded. She agreed to and allowed video recording of the goals at T1 and T2. At T3 (3-month follow-up), she performed one goal (cracking an egg without eggshells) and allowed this performance to be video recorded. She did not engage in re-rating her goal performance on the COPM. As she saw goal progress and built trust with the occupational therapist, she engaged more in sessions over time. As Kate illustrates:

*It took a bit of time to get her to cooperate, and it sometimes felt that she wasn’t really going to do anything… So you sort of have to do something sensory first [before knitting], like the egg or the bike or this hammock or something. And then and then she might like, agree to do some knitting, but just that repetition* (Kate).

Kate also acknowledged that her daughter acquired bike riding quickly and knitting was a slower process. As such, Sarah experienced some frustration with knitting, as described by Kate:

*Like if she saw more progress a little bit quicker, it would have turned a bit more. Yeah, but the knitting’s really slow, so it’s well for a beginner as you don’t see fast enough progress and I guess it’s a bit off putting for her* (Kate).

Participant responses to CO-OP, such as reporting feeling stressed, frustrated, fatigued, experiencing low mood, disengagement and being video recorded, were managed throughout the study ethically and responsively. Mechanisms were in situ, as part of our ethics approval to monitor and respond to participant responses to CO-OP. This included: the CO-OP therapist allowed children to decline activities, adjusted expectations of practicing the three goals and checked in with caregivers about children’s and their own well-being. Children were offered play and snack breaks between activities when required to restore energy and ability to engage. Regular supervision with the research team throughout data collection ensured that all reasonable steps were taken to manage and support participant responses during CO-OP. No adverse events occurred.

#### 3.4.1. PRIME-O

**Figure 3:**
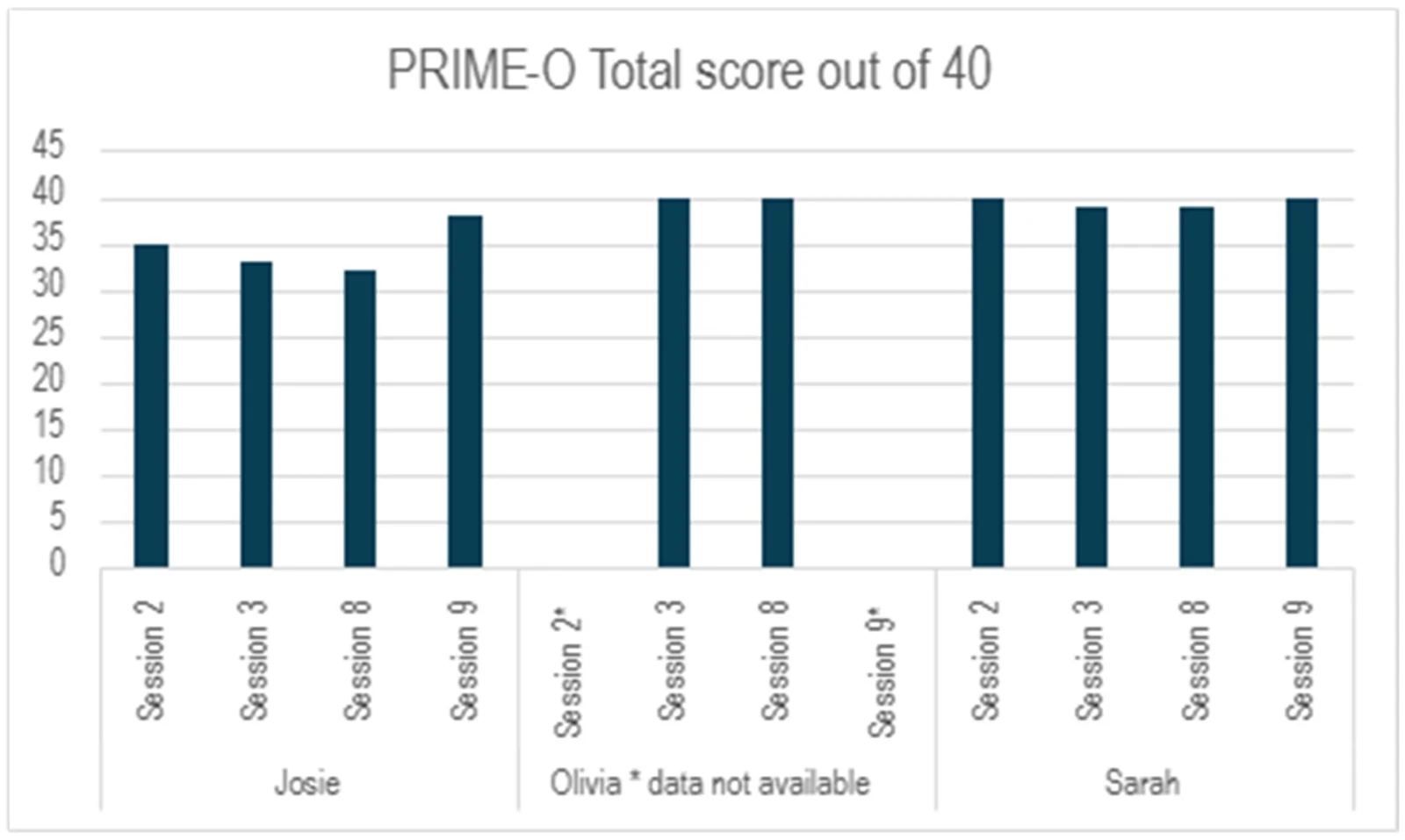
Participant results on the PRIME-O. * data not available.

Participant results on the PRIME-O were positive, and all three child participants engaged in sessions with the therapist. Josie’s PRIME-O scores ranged from 32 to 38 out of 40 (Figure 3). PRIME-O data for Olivia’s S3 and S8 were high (40/40). PRIME-O data was missing in S2 and S9 (Figure 3). PRIME-O scores for Sarah were high across all four sessions ranging from 39 to 40 out of 40 (Figure 3).

#### 3.4.2. CO-OP Fidelity

Fidelity scores across participants and sessions (S1, S6 and S8) were very good. Within-session scores ranged from 76% to 84%. Across-session scores ranged from 80% to 93%. General fidelity scores were good to high ranging from 78% to 97% (Supplement File S6).

### 3.5. Expansion

All three children identified three goals and rated their perceived performance and satisfaction using the COPM at the three timepoints (T1, T2 and T3). The children rated their own performance lower than their caregivers’ performance and satisfaction ratings. During CO-OP practice, children used the primary metacognitive strategy (Goal, Plan, Do and Check) and created domain-specific strategies with therapist and caregiver support. By the 6-month follow-up (T3), Josie had forgotten aspects of this, and Olivia was not implementing CO-OP. At the 3-month follow-up (T3), Kate commented that Sarah continued to use the metacognitive strategy and recognised that Sarah planned and checked activities. She said:

“*So if we’re finding that there is something that she needs to or wants to do and then she’ll think about how she’ll do it*” *and* “*well she checks, I ‘spose she checks to see whether she’s done a good job*.”

Regarding Goal, Plan, Do and Check specifically, Kate reflected that as a family “*we don’t remember to use the language*” yet recognised that Sarah was using parts of the strategy in her daily life.

All children progressed towards independence in their performance goals to varying degrees. Caregivers noted positive differences in their child’s participation and performance after CO-OP, as noted by Helen and Kate:

*But because she has been doing it, she has been doing it regularly for now how many weeks has it been now, she’s not, she just opens the fridge now* (Helen).

*She just got it [bike riding without training wheels] all of a sudden, she got inspired and because she started to see that she was making progress, like turning corners without a problem* (Kate).

### 3.6. Limited Efficacy

Results of pre- and post-COPM and PQRS-G scores are provided in Table 4, Table 5 and Table 6 for Josie, Olivia and Sarah, respectively. At T2, Josie rated meaningful changes in performance and satisfaction on the COPM for all three practiced goals and in performance on one goal not practiced (washing hair). At T3 (6-month follow-up), she rated meaningful changes in performance for all three practiced goals and one of her goals not practiced (hair washing). Helen rated meaningful changes in Josie’s performance on all three practiced goals at T2 and at T3. At T2, Josie made clinically meaningful changes in her PQRS-G scores on two out of three practiced goals (tying shoelaces and retrieving/replacing items to/from the fridge). Her goal of spreading butter with a knife decreased at T2 and T3 compared to T1. This was due to her scoring highly at T1. Other reasons for the change were not clear. PQRS-G scores for her two non-practiced goals could not be obtained as they were not recorded (Table 4).

Olivia’s self-ratings on the COPM for performance and satisfaction of her practiced goals were variable. At T2, she rated negative changes on performance and satisfaction for her goal of keeping her room tidy. Olivia rated a clinically meaningful improvement in satisfaction for her goal of keeping her school bag tidy and improvements in performance and satisfaction for her goal of writing neatly on lines. At T3 (6-month follow-up) she maintained clinically meaningful satisfaction ratings for writing neatly on lines but rated no changes in her perceived performance from T1. At T2, her father, Greg, rated clinically meaningful changes in performance and satisfaction for one of her three practiced goals (keeping her school bag tidy). He also rated negative changes in keeping her room tidy and did not rate meaningful changes in her goal of writing neatly on lines. At T3, Greg rated a clinically meaningful improvement in performance for keeping her room tidy. He did not rate meaningful changes in her goal of writing (Table 5). PQRS-G scores were not available for two of Olivia’s goals due to the nature of the goals (keeping her room tidy and keeping her school bag tidy). Olivia did make changes at T3 (6-month follow-up) on the PQRS-G for her goal of writing neatly on lines. Video data at T2 for writing was not obtained and thus could not be scored.

At T2, Sarah self-rated clinically meaningful changes in performance and satisfaction on one of her practiced goals (riding a bike without training wheels). At T2, Kate rated positive changes on performance and satisfaction for Sarah’s goal of riding a bike without training wheels. She rated positive changes in Sarah’s performance for cracking an egg without eggshells and positive changes in satisfaction for knitting a square. At T3 (3-month follow-up) Kate rated clinically meaningful changes on performance and satisfaction for two of Sarah’s goals (riding a bike without training wheels and cracking an egg without eggshells). Sarah made positive changes in goal quality at T2 on two of her three goals (cracking an egg without eggshells and knitting a square). Her goal performance quality was maintained at the 3-month follow-up (T3) for cracking an egg without eggshells. Her other goal performance could not be rated at T3. As Sarah could not engage in goal setting at T3, her COPM scores were not rated (Table 6).

Minimal change was detected in the home frequency of participation using the PEM-CY [33] (Supplement File S7). Despite limited to no changes in PEM-CY data, all caregivers reported changes in their child’s participation in leisure and self-care occupations. For example, Greg reported, “*When we’re playing UNO, [Olivia]’s sort of a bit better at keeping up with the game*” and noted, “*she is more independent now. She only needs little reminders*.” Kate described the impact of Sarah riding her bike without training wheels:

*And we and we’ve got the support workers on it as well and they all go to the park, and she rides a bike. They go, the support worker goes on [brother]’s scooter, [brother] takes the other scooter and they just all go together, so it’s really nice* (Kate).

Helen reported the following observation:

*I just said quick, you did it the day before and, she did, she used her shirt to grab it [milk carton in fridge] and take it out. It wasn’t a big deal, but it’s a way that she worked out how to do it. So, she put a plan, she thought of something… She put a plan into action and she did it….Whereas in the past she would not have done that* (Helen).

### 3.7. Demand

Enrolment was below the planned target of six to ten children. Four participants enrolled, and all four were eligible to participate. Retention was high with three out of the four participants remaining in the study.

### 3.8. Study Design

This study design was feasible regarding recruitment and participation rates. Not all data collection was feasible (Table 3).

#### Protocol Changes

Due to the exploratory nature of this study, there were minor changes to our planned protocol. These included an additional goal setting session for Sarah, replacement of home practice logs with informal discussions, delayed follow-up and incomplete outcome measures (by caregivers and children). These changes were made depending on participant engagement in sessions, requests not participate in some goal practice and to have sessions recorded and availability.

## 4. Discussion

The findings of this study provide preliminary support for the feasibility of the CO-OP approach for children with FASD and motor coordination difficulties, although results were limited due to the small sample size. With a sample of only three participants, all had to endorse the criterion to achieve the 80% threshold for it to be considered met. Of the six feasibility criteria, two were met (acceptability and expansion), and four were not met (practicality, implementation, limited efficacy and demand). Of the study design feasibility criteria, two were met (recruitment rate and participation rate) and one was not met (data collection). Several key findings were identified from the study results. These are reviewed and discussed in relation for feasibility.

### 4.1. Acceptability

This study captured the experiences of CO-OP from both caregivers and children with FASD. While parents, adolescents and young adults have identified CO-OP to be an acceptable, feasible and effective approach [37], little is known about the participation experiences of children younger than 16 years [37,38]. Parents’ experiences of participating in CO-OP as reported by Gharebaghy et al. [37] provided key learnings for clinicians to enhance support to caregivers during CO-OP to increase implementation, transfer and generalisation. However, there is a knowledge gap in understanding children’s experiences of CO-OP, which is vital to maximise their participation and involvement in goal achievement and provide practitioners with further guidance to maximise its application.

In our study there were mixed reactions to CO-OP by the caregiver and child participants, however all found it acceptable. All caregivers described aspects of the approach that they found acceptable and satisfactory, and they all requested more sessions to support their child’s goal practice and achievement. One child described the approach as “stressful” (Josie) to plan, problem solve and make decisions. She experienced frustration when performing and practicing her goals which limited her motivation to practice. Her caregiver (Helen) expressed frustration with learning to implement the CO-OP approach with her daughter as she felt there were faster ways of achieving goals such as directly telling her daughter how to do the task. This is a common experience of caregivers when they are new to CO-OP [39]. In a study by Dietrich et al. [39] caregivers identified difficulties in knowing how to guide their children using guided discovery without feeling frustrated or giving them the answer. They needed to learn a new way of guiding their children without doing things for them. Likewise, in our study, Helen learned to use guided discovery with Josie with the CO-OP therapist and gained reassurance that CO-OP could help Josie retain how to perform her goals. Young adults with cerebral palsy and spina bifida provide insight into the challenges they encountered when learning CO-OP [38]. They identified that CO-OP was a different way of learning and, despite finding it physically and mentally straining, they found it worthwhile. Using their new problem-solving skills in everyday life, they were more willing to try new activities that they had previously avoided.

Another child (Sarah) also experienced some frustration with the slow progress of her knitting goal and did not continue to practice knitting after CO-OP. Despite feeling frustrated with the slow progress, her caregiver reported that Sarah enjoyed and looked forward to the CO-OP sessions as she got to practice and achieve her other goals. All three children felt that CO-OP was useful for children with FASD to help them acquire skills and saw some benefit from participating in CO-OP.

### 4.2. Practicality (Ease of Use)

Although all caregivers valued CO-OP and the choice it gave their children in identifying their own occupational performance goals, they encountered challenges implementing home practice with their children outside of sessions. This included child factors and family involvement. Child factors included motivation to practice and perceived goal success, emotional responses to practicing their goals, remembering to use metacognitive strategies and physical and cognitive fatigue. Home practice was also influenced by family involvement and the embedding of practice into daily routines. For example, Olivia practiced tidying her bedroom weekly with her stepmother and Sarah practiced knitting during bedtime with her caregiver. When Sarah’s same-aged cousin participated in a CO-OP session and identified that Sarah could ride a bike better than her cousin, this increased Sarah’s motivation to practice bike riding. Context, both social and routine-based, are known to influence children’s participation [40] in occupations.

Motivation has been described by others as an essential component of the success of CO-OP for children with cerebral palsy and other disabilities [41,42,43]. Research supports the use of child-identified goals in CO-OP to improve their self-efficacy, cooperation and motivation [7,37,44,45]. In our study, caregivers reported that child-chosen goals were meaningful and supported their child’s motivation. Home practice of identified goals outside of CO-OP sessions was dependent on the child’s motivation and perceived efficacy of performance as well as caregiver availability to support practice. Although not all goals were practiced every week between sessions, at least one was practiced weekly. As children became more competent in their goal performance, they increased their engagement in and outside of the CO-OP sessions.

All children experienced challenges with remembering and using the global strategy (Goal, Plan, Do, Check) outside of sessions and at follow-up. Memory difficulties are commonly impacted by prenatal alcohol exposure [46], and children with FASD experience more significant difficulties than children with ADHD alone [47]. Repetition, routine, structure and supervision are some of the Eight Magic Keys recommended strategies for people with FASD to support memory, learning and daily participation [48] and are supported by the CO-OP approach.

The metacognitive strategy use was facilitated by the caregiver’s capacity to support and embed home practice into daily routines. All caregivers suggested that having more than 10 CO-OP sessions could better facilitate practice and goal achievement and support their ability to help their children use CO-OP. Regular practice is important to make substantial changes in the motor performance of children with DCD [49] and can help to reinforce the cognitive, problem-solving approach. Araujo et al. [50] have explored the efficacy of the dual approach of CO-OP with occupational performance coaching (OPC) [51] for children with DCD. In their study, caregivers received four additional 60 min coaching sessions in groups every other week along with CO-OP [50]. The authors found that there were no additional benefits to using OPC and it did not result in superior gains in occupational performance, participation or motor performance of children with DCD. Gharebaghy et al. [37] investigated Iranian mothers’ experiences of being involved in the transfer of the CO-OP approach. They identified that caregivers benefit from explicit education and coaching to use guided discovery with their child. In their study, mothers reported low confidence in learning and applying CO-OP principles in new situations without the CO-OP therapist. They also found supporting their child’s behavioural challenges during implementation challenging. The mothers benefited from CO-OP therapist support using step-by-step teaching of the principles to understand and apply the problem-solving process and guided discovery themselves [37]. The authors reported that for caregivers, “knowing about the CO-OP approach and observing the sessions are not sufficient” [37] (p. 8) and more hands-on practice in implementing strategies with CO-OP coaching was required. More explicit coaching, education and support may have supported the caregivers in our study to more readily adopt the CO-OP principles and support home practice. Further research is required to investigate how caregivers respond to hands-on practice in implementing CO-OP and coaching from the CO-OP therapist.

### 4.3. Implementation

This feasibility study was implemented with some changes to the study protocol to reflect participant needs. The study was implemented with high rates of CO-OP intervention fidelity. The protocol was implemented by one certified CO-OP therapist (CH) who travelled to the participant’s homes. This travel was grant funded, and attendance was high, with all 10 sessions being completed for each participant.

As a key feature of the CO-OP approach, client-centeredness was achieved by using self-selected goals [7]. Caregivers valued this approach and recognised that it helped motivate their child because the goals directly impacted their daily living. Similar research in other occupational therapy literature has found that well-established child-centred approaches to facilitate power-sharing and empowerment of children through goal setting enhance child motivation [52]. This study investigated caregivers’, children’s and occupational therapists’ experiences of children’s participation in goal setting. Semi-structured interviews were conducted with participants and information from children was elicited using drawing, role play and photo elicitation techniques. Interviews were conducted in family homes with parents present during child interviews [52]. Similar to the caregivers’ perspectives in our own study, one caregiver valued child-chosen goal setting “because it affects them 100 percent” [52] (p. 10). Children also valued being included in their decision-making of the CO-OP approach in our study despite mixed feelings about practicing challenging tasks.

The implementation of goal setting for two children in our study (Olivia and Sarah) did not go as planned. For Olivia, she chose to discontinue her goal of tidying her school bag in S7. Sarah required two sessions rather than one to identify three goals for CO-OP (S1 and S2). She benefited from extra time to build rapport with the CO-OP therapist, help her understand the goal setting process and explore her desired goals.

Children’s engagement in sessions varied across sessions, as evaluated on the PRIME-O [28] and as evident in field notes and video recordings. It was dependent on personal factors including building trust with the CO-OP therapist, understanding of CO-OP expectations, emotional readiness to engage, persistence, cognitive and physical effort and caregiver availability to support the children’s co-regulation and problem solving. Children’s perceived success influenced their willingness to perform their goals during and outside of sessions. For example, once Sarah gained confidence in her ability to ride her bike without training wheels, she rode her bike every day after school. By contrast, she found knitting, much more challenging to achieve and was not motivated to continue with it after the CO-OP sessions.

Children’s emotional response and readiness to engage in CO-OP have also been identified by caregivers of children and youth with executive functioning deficits after acquired brain injury [39]. Caregivers have identified that their child’s “emotional frame of mind (enthusiasm, cooperativeness, level of attention)” (p. 6) influenced the success of their problem-solving approach. Like our study, the caregivers in the study by Dietrich et al. [39] also found it challenging to implement home practice with their children when their children were tired, irritated and not ready to engage. Sarah was one participant in our study who required extra time to build trust and actively participate in the problem-solving approach. Her caregiver, Kate, was present during sessions and supported Sarah to attempt and persist with her goals. At follow-up, Sarah minimally engaged in the session and demonstrated one goal performance (cracking an egg without eggshells). She could not re-rate her goal performance and satisfaction on the COPM even with visual supports (e.g., a picture of a ladder and smiley faces).

Using the CO-OP approach across 10 × 30–60 min sessions, all children explored, practiced and acquired specific strategies to achieve goal performance for at least one goal facilitated by the CO-OP therapist. The seven key features of the CO-OP protocol (child identified occupational performance goals, dynamic performance analysis, cognitive strategy use, guided discovery, enabling principles, caregiver involvement and intervention format) [7] were implemented and were essential to support each child’s understanding of therapy expectations and involvement, as well as goal performance success. CO-OP aligns with the widely recommended Eight Magic Keys approach for people with FASD [48]: concrete, consistent, repetitive, routine-based, simple, specific, structured, and supervised.

The CO-OP protocol and the therapist’s implementation skills and flexibility supported the children’s problem solving and goal performance and increased caregiver confidence to support their children in using CO-OP strategies.

### 4.4. Limited Efficacy and Expansion of CO-OP to Children with FASD

#### 4.4.1. Occupational Performance

All three children in this study made clinically meaningful gains (i.e., ≥2 points) on at least one practiced goal as rated by caregivers and children on the COPM and on their goal performance, measured on the PQRS-G (i.e., ≥3 points) after CO-OP (T2). At follow-up (T3), all participants maintained quality of performance on at least one goal. Child and caregiver ratings on the COPM varied. Generally, caregivers reported maintenance of satisfaction and performance on the COPM at T3. Greg, Olivia’s father, reported improvement in performance on Olivia’s goal of keeping her room tidy but no changes in satisfaction. He did not rate changes in performance or satisfaction of ≥2 points with her goal of writing neatly on lines; however, Olivia rated an improvement in her satisfaction with writing. This suggests that CO-OP may support the maintenance of some goals rated by children and caregivers and performance measured by the PQRS-G for these participants.

Other studies with children similar to those in this study who had executive functioning deficits, two of whom also had a diagnosis of ASD, found similar findings. There is a growing body of evidence showing that CO-OP is effective in supporting occupational performance goals for children with executive functioning deficits, including those after acquired brain injury [9,10,39], those with ADHD [53,54,55,56] and those with autism [57], although they take longer to learn to use the metacognitive strategies and do not always retain these compared to children with DCD.

Implicit motor learning strategies may benefit children with more limited verbal skills. In a study by Izadi-Najafabadi et al. [58], children with autism (aged 7–11 years) were tested on a computer-based serial reaction time task (SRTT) and compared with non-autistic peers. The former had impaired explicit motor learning, but intact implicit motor learning. The authors proposed that providing more implicit motor learning strategies by helping children to discover their own domain-specific strategies (for example, feel the movement and body position) may be more beneficial to autistic children than explicit strategies (such as verbalising planning) during CO-OP [58]. This is consistent with the two participants with co-occurring autism who found verbalising plans difficult.

For some goals that were less motivating, such as Olivia’s goal of tidying her bedroom, or perceived as challenging, such as Sarah’s knitting goal, children tended to negatively rate their performance and satisfaction post-intervention. Greg was less involved in his daughter’s CO-OP and rated lower levels of performance and satisfaction for her goals immediately after the CO-OP at T2. At T3 (6-month follow-up), he rated greater changes in her goals. It may be that it took additional time, and more availability of the caregiver, to detect changes in his daughter’s goals. Children rated their goal performance and satisfaction differently from their caregivers. Likewise, in a case study of two children by Rodger et al. [45], children rated their goal performance differently from their caregivers and one participant (Thomas) decreased his satisfaction rating with using cutlery due to reduced motivation towards the goal. From a children’s rights perspective, O’Connor et al. [52] acknowledge that children have different views from adults and experience situations differently and adult proxies cannot account for children’s needs. Thus, it is important to include children’s own self-ratings in evaluation.

#### 4.4.2. Participation

Our study showed no significant changes in children’s frequency of participation at home, school or in the community on the PEM-CY [33] (Supplement File S7). A similar finding was made by Araujo et al. [50] who investigated the home and community indices of the PEM-CY to evaluate participation outcomes following CO-OP in 22 children with DCD. The authors questioned the responsiveness of the PEM-CY to detect change in participation in children with DCD [50]. Caregivers in our study did not find the PEM-CY satisfactory as an outcome measure to complete. Most caregivers (2/3) described it as repetitive, and one participant did not complete the full questionnaire at T3. We identified changes in children’s participation in leisure and self-care activities through interview feedback from caregivers. Setting participation goals with children and conducting in-depth interviews post CO-OP may provide a useful way to understand participation changes rather than relying on the PEM-CY.

### 4.5. Demand

The demand for CO-OP among children with FASD in the ACT and surrounding NSW could not be calculated based on recruitment rates. Enrolment was below the planned target. Despite a relatively high FASD prevalence and growing public awareness of FASD in Australia, there is no public diagnostic clinic in the ACT, which may have prevented referrals in the ACT region. All children in the study were referred from a NSW-based FASD diagnostic clinic. Recruitment occurred over a 22-month period, and all efforts to recruit participants were exhausted.

### 4.6. Study Design

The study design criteria were feasible; however, there were several changes made to the study protocol to accommodate participant engagement and needs. The use of independently rated PQRS-G, child- and caregiver-rated COPM scores, supported by qualitative interviews and field notes, were effective in capturing participant outcomes. Home programme logs were changed to home practice discussions due to child- and caregiver-reported barriers to implementing home practice. The casual discussions, often conducted off video recording, facilitated a less judgement-based review of home practice and, more importantly, a discussion about how to overcome the barriers. The use of field notes was essential to record sessions, goal performance and home practice, particularly when children declined to have sessions video recorded. This limited the capture and subsequent PQRS-G rating of goal performance. The timing of follow-up was not completed as planned at 3 months. This was due to difficulty reconnecting with two families, and instead the follow-up for Josie and Olivia occurred at 6 months. These protocol changes should be considered for future implementation studies of CO-OP with children with FASD and their families.

### 4.7. Study Strengths and Limitations

This is the first study to investigate the feasibility of the CO-OP approach for children with FASD and motor coordination difficulties. It has several limitations which limit the generalisability of the findings to other children with FASD. First, although this study presents a case series of three female children aged 11 and 12 years with FASD, they had varied neurocognitive abilities and co-occurring diagnoses, which were representative of children with FASD. Caregivers involved were biological and adopted parents and both genders were represented. Due to time and budget restrictions and a paucity of available participants, further recruitment was not possible. The sample size is small, and findings are restricted to the experiences and results of these participants. Second, interviews were conducted and analysed by the therapist who delivered CO-OP, which may have influenced the findings. Caregivers and children requested to be interviewed by the CO-OP therapist, rather than an independent interviewer, and were candid in their responses. Rigour in the thematic analysis was ensured by spending extensive time with the data, having other authors review the analysis, and maintaining detailed coding logs. Third, we made changes to our research protocol due to changes in children’s and caregiver’s needs. This represents a pragmatic challenge for research when it is embedded in participant’s home environments and everyday lives. Future research should include a larger sample and a broader range of ages and genders among children with FASD to better understand the potential effects of CO-OP in this population.

### 4.8. Implications for Practice

First, this study suggests that CO-OP may help improve occupational performance and participation in children with FASD and motor coordination difficulties. The client-centred approach of CO-OP [7] facilitates children’s identification of their own goals for therapy and involvement in their therapy decision-making. This is the first study reported to have enabled children with FASD to identify their own goals for therapy [6]. The CO-OP protocol and therapist can support children to practice their goal performance and identify domain-specific strategies to acquire goal performance. The repetition of practicing three goals in each session, the use of enabling principles and guided discovery principles [59] support learning and skill acquisition in children with FASD during CO-OP sessions.

Second, this study demonstrates that children with FASD can identify meaningful occupational performance and participation goals with caregiver and therapist support. When implementing CO-OP with children with FASD, goal setting may be further aided using visual supports such as the PEGS [22] and visual analogue scales to support COPM ratings [24] and by allowing more time. Other important factors for successful goal setting include allowing more than one goal-setting session to build rapport and clarify expectations and ensuring the CO-OP therapist fosters trust and a safe environment for children and caregivers [60]. This is particularly essential for using trauma-informed practice, as many children with FASD have experienced trauma [61].

Third, children with FASD may benefit from more than 10 sessions to implement CO-OP. This could include additional sessions up to 14 [9], further caregiver training in understanding how to implement CO-OP [37], and additional check-ins with additional sessions at three months post-CO-OP. Children with FASD often have impaired memory, executive functioning challenges and require repetition to acquire skills [1,46]. The caregivers in our study advocated for more CO-OP sessions as it took time to build rapport with their children and for their children to experience success performing their goals.

Finally, caregiver involvement is essential to support successful implementation [37]. Careful consideration of caregiver and family capacity and a thorough explanation of the CO-OP approach are required before implementation. Providing coaching, explicit, step-by-step instruction about how to support guided discovery and hands-on practice during sessions may enhance caregiver confidence.

## 5. Conclusions

This descriptive case series provides some preliminary support for the feasibility of CO-OP for children with FASD to address occupational performance and participation through self-chosen goals. Despite challenges reported by children and caregivers in participating in CO-OP and varied goal achievement results, children and caregivers found CO-OP acceptable. CO-OP presented an opportunity for children with FASD to set their own therapy goals and actively participate in the intervention. These early findings highlight the importance of caregiver and family involvement, taking a flexible and trauma-informed approach, allowing children and caregivers time to build rapport, trust and understanding of the CO-OP process. Additional sessions for goal setting and intervention suggested by the caregivers in this study may support goal achievement. It can be concluded that CO-OP appears feasible in this small-scale case series and warrants a larger pilot or randomised controlled trial with protocol modifications to further test feasibility and effectiveness, acknowledging that further testing may be impacted by protocol changes and recruitment challenges.

## Figures and Tables

**Table 1:** Timeline of sources of data collected.

	T1 Baseline	S1	S2	S3	S4	S5	S6	S7	S8	S9	T2 S10	T3 Follow-Up
Demographic questionnaire	*											
Vinelands Adaptive Behaviour Scale Third edition domain form (VABS-3)	*											
BruininksyOseretsky Test of Motor Proficiency Short Form second edition (BOTSF-2)	*											
Field notes & reflexive journal	*	*	*	*	*	*	*	*	*	*	*	*
Audio/video session recordings	*	*	*	*	*	*	*	*	*	*	*	*
Canadian Occupational Performance Measure (COPM)	*										*	*
CO-OP Fidelity Checklist		*					*			*		
Performance Quality Rating Scale Generic (PQRS-G)	*										*	*
Participation in environment Measure for Children and Youth (PEM-CY)	*										*	*
Parents as Partners in Intervention Satisfaction (PAPI III)											*	
Home practice log discussions		*	*	*	*	*	*	*	*	*	*	
Pediatric Rehabilitation Intervention Measure of Engagement—Observation (PRIME-O)			*	*					*	*		
Child & caregiver interview											*	*

T1 timepoint 1 prior to CO-OP; S1–S10 CO-OP S1 to S10; T2 timepoint 2 during S10; T3 timepoint 3 months post S10 for one family; 6 months post S10 for two families. * = time point when data was collected.

**Table 2:** Child and caregiver characteristics.

Child Characteristics	Josie *	Olivia *	Sarah *
Age	12	12	11
Gender	Female	Female	Female
Ethnicity	Caucasian	Caucasian	Vietnamese-Loatian
Siblings	0	2	2
Age of FASD diagnosis	7 years	11 years	8 years
Other diagnoses	None	Autism Depression	Autism ADHD
Neurodevelopmental domains assessed as severe impairment	4 Motor skills Academic achievement (maths) Attention Affect regulation	5 Motor skills Academic achievement (maths, spelling) Executive functioning Affect regulation Social skills	7 Motor skills Language Academic achievement Memory Attention Executive functioning Adaptive behaviour
Other therapy	Psychology, Educational support	Occupational therapy Psychology	On a break from occupational therapy
Medication	None	Anti-depressant	For anxiety and ADHD
VABS3 ABC Communication Daily Living Skills Socialisation	7th percentile 5th percentile 7th percentile 21st percentile	5th percentile 7th percentile 9th percentile 7th percentile	2nd percentile 5th percentile 2nd percentile 3rd percentile
BOT2 SF	12th percentile	4th percentile	8th percentile
DCD-Q TMS	38/75	30/75	46/75
**Parent characteristics**	Helen *	Greg *	Kate *
Gender	Female	Male	Female
Relationship to child	Adopted parent	Biological father	Adopted parent
Level of education	University	Unknown	Post-graduate
Employment status	Full-time employed	Full-time employed	Part-time employed

* Pseudonyms used. VABS3 = Vinelands Adaptive Behaviour Scale 3rd edition. ABC = Adaptive Behaviour Composite Score. BOT2 SF = Bruininks Oseretsky Test of Motor Proficiency Short Form. DCD-Q TMS = Developmental Coordination Questionnaire Total Motor Score.

**Table 3:** Feasibility criteria results (*n* = 3).

Feasibility Domain	Feasibility Question	Data Collected	Findings
Feasibility of CO-OP	*Acceptability:* Is CO-OP acceptable to participants?	Caregiver semi-structured interviews Child interviews PAPI III	CO-OP was found to be acceptable to children and caregivers. Two of the three children (66%) reported enjoyed doing CO-OP and all children (100%) recommended CO-OP for other children with FASD to help them do activities better. All caregivers (100%) reported satisfaction with CO-OP during interviews. All caregivers reported satisfaction with CO-OP on the PAPI III and feeling like a partner and working with their child at home (100%). **MET for caregivers, NOT MET for children**
	*Practicality (Ease of use):* Do participants find CO-OP easy to use?	Caregiver semi-structured interviews Child interviews Home practice logs	Caregivers and children did not find CO-OP easy to implement without the CO-OP therapist during home practice. Caregivers found it challenging to motivate and engage children and fit in practice to busy family lives. All children practiced at least one goal once a week. Only one child practiced a goal daily (Olivia) by writing daily at school. No child reported twice weekly practice of each goal. **NOT MET**
	*Implementation:* Is the CO-OP implemented as per the protocol?	CO-OP Fidelity Checklist PRIME-O/engagement Completed measures and all protocol data. Video data Field notes and reflective journal Early withdrawal survey Attendance	CO-OP was implemented as per the protocol. Very good fidelity scores were rated for all participants across sessions (76–84%). The PRIME-O scores for all three participants (100%) were high between 32 and 40/40. Outcome measures (Most COPM, PQRS-G, PAPI III) were completed by participants. The PEM-CY was not completed by one caregiver (Greg), and one child participant (Sarah) could not engage in re-scoring her COPM at follow-up. All participants attended every session, although two participants (Josie and Sarah) did not engage in all sessions. Josie practiced her goals on 7/10 sessions. Sarah practiced cracking eggs every session, knitting on 6/10 sessions and riding her bike without training wheels on 3/10 sessions. At follow-up Sarah practiced 1/3 goals (cracking eggs). **NOT MET**
	*Expansion:* Can children with FASD use CO-OP to address occupational performance and participation goals?	COPM performance and satisfaction scores reported by children and caregivers PQRS-G rating scores Video data Field notes and reflective journal	All three children (100%) identified three goals for CO-OP and all three (100%) made clinically meaningful changes on at least one practiced goal immediately following CO-OP (T2) as measured by the COPM and PQRS-G. **MET**
	*Limited efficacy testing:* Does CO-OP improve child and caregiver-rated performance and satisfaction scores on identified goals, quality of goal performance and participation?	COPM performance and satisfaction scores reported by children and caregivers PQRS-G scores PEM-CY scores	Increased scores were observed for COPM and PQRS-G for all participants (100%) on at least one practiced goal at T2. At T3 all participants (100%) maintained quality performance on at least one goal measured by the PQRS-G. Changes were not observed on the PEM-CY for children however caregivers reported changes in their child’s participation in leisure, self-care and learning activities. **NOT MET**
	*Demand:* How many participants enrol in this study?	Enrolment records	Four children out of an expected six to ten (40%) participated. **NOT MET**
Feasibility of study design	*Recruitment rate:* How many participants screened are eligible to participate?	Attendance records Field notes	4/5 children (80%) who were screened were eligible to participate in this study. **MET**
	*Participation rate:* Do all eligible participants who agree to participate complete CO-OP?	Attendance records	All children recruited (3/3), participated in this study for all 10 CO-OP sessions. **MET**
	*Data collection:* Can planned data be collected a baseline (T1), post-training (T2) and 3 months’ post training (T3)?	Completed BOT2-SF, VABS3, COPM, PQRS-G, PEM-CY, PAPI and demographic questionnaires. Field notes	Most data were collected as per protocol. All children and caregivers (100%) completed pre-outcome measurements (T1) and post-outcome measurements (T2). 2 out of 3 children (66%) participated in follow-up outcome measure appointment (T3). One caregiver (Greg) did not complete all sections of the PEM-CY. Sarah could not engage in self-rating her COPM scores at follow-up. Demographic, VABS3 and PAPI questionnaires were completed by all caregivers (100%). All children were screened using the BOT2-SF (100%). **NOT MET**

**Table 4:** Josie’s results on the COPM and PQRS-G.

Goal	Pre-Intervention Score T1 Out of 10	Post-Intervention Score T2 Out of 10	Change Score T2 − T1	Follow-Up (at 6 Months) T3 Out of 10	Change Score T3 − T1
**Spread butter with a knife** *					
COPM performance C	2	6	4 ^#^	5	3 ^#^
COPM satisfaction C	2	4	2 ^#^	5	3 ^#^
COPM performance P	3	7	4 ^#^	5	2 ^#^
COPM satisfaction P	2	8	6 ^#^	5	3 ^#^
PQRS-G	8	5	−3 ^###^	4	−4 ^###^
**Tie shoelaces** *					
COPM performance C	1	8	7 ^#^	3	2 ^#^
COPM satisfaction C	1	6	5 ^#^	1	0
COPM performance P	1	7	6 ^#^	8	7 ^#^
COPM satisfaction P	1	8	7 ^#^	7	6 ^#^
PQRS-G	3	8	5 ^##^	6	3 ^##^
**Retrieve/replace items to/from fridge** *					
COPM performance C					
COPM satisfaction C	2	6	4 ^#^	4	2 ^#^
COPM performance P	2	4	2 ^#^	4	2 ^#^
COPM satisfaction P	3	9	6 ^#^	7	4 ^#^
PQRS-G	3	9	6 ^#^	9	6 ^#^
**Wash hair**					
COPM performance C	3	6	3 ^#^	6	3 ^#^
COPM satisfaction C	5	5	0	5	0
COPM performance P	5	7	2 ^#^	5	0
COPM satisfaction P	5	8	3 ^#^	7	2 ^#^
PQRS-G	NS	NS	NS	NS	NS
**Manipulate soft plastics**					
COPM performance C	1	1	0	1	0
COPM satisfaction C	1	1	0	1	0
COPM performance P	1	1	0	1	0
COPM satisfaction P	1	1	0	1	0
PQRS-G	NS	NS	NS	NS	NS

* Practiced goals; ^#^ clinically meaningful COPM improvement in score of 2 or more; ^##^ clinically meaningful PQRS-G improvement in score of 3 or more; ^###^ clinically meaningful PQRSG-G decline in a score of 3 or more C = child-rated; P = caregiver-rated; COPM = Canadian Occupational Performance Measure; PQRS-G = Performance Quality Rating Scale Generic; NS = not scored.

**Table 5:** Olivia’s results on the COPM and PQRS-G.

Goal	Pre-Intervention Score T1 Out of 10	Post-Intervention Score T2 Out of 10	Change Score T2 − T1	Follow-Up at 6 Months T3 Out of 10	Change Score T3 − T1
Keep room tidy *					
COPM performance C	5	4	−1	5	0
COPM satisfaction C	7	2	−5	6	−1
COPM performance P	5	3	−2	8	3 ^#^
COPM satisfaction P	7	3	−4	8	1
PQRS-G	NS	NS	NS	NS	NS
Keeping school bag tidy *					
COPM performance C	4	4	0	NS ^$^	NS ^$^
COPM satisfaction C	1	4	3 ^#^		
COPM performance P	4	10	6 ^#^		
COPM satisfaction P	4	7	3 ^#^		
PQRS-G	NS	NS	NS		
Writing neatly on lines *					
COPM performance C	6	9	3 ^#^	6	0
COPM satisfaction C	3	10	7 ^#^	5	2 ^#^
COPM performance P	7	8	1	8	1
COPM satisfaction P	8	8	0	8	0
PQRS-G	4	NS	NS	7	3 ^##^
**Reading long words**					
COPM performance C	2	6	4 ^#^	7	5 ^#^
COPM satisfaction C	3	8	5 ^#^	10	7 ^#^
COPM performance P	5	4	−1	7	2 ^#^
COPM satisfaction P	7	4	−3	7	0
PQRS-G	9	3	−6	NS	NS

* Practiced goals; ^#^ clinically meaningful COPM improvement in score of 2 or more; ^##^ clinically meaningful PQRS-G improvement in score of 3 or more; C = child-rated; P = caregiver-rated; COPM = Canadian Occupational Performance Measure; PQRS-G = Performance Quality Rating Scale Generic; NS = not scored. NS ^$^ = chose to discontinue working on this goal.

**Table 6:** Sarah’s results on the COPM and PQRS-G.

Goal	Pre-Intervention Score T1 Out of 10	Post-Intervention Score T2 Out of 10	Change Score T2 − T1	Follow-Up at 3 Months T3 Out of 10	Change Score T3 − T1
Ride bike without trainers *					
COPM performance C	1	7	6 ^#^	NS	NS
COPM satisfaction C	1	6	5 ^#^	NS	NS
COPM performance P	1	8	7 ^#^	8	7 ^#^
COPM satisfaction P	1	8	7 ^#^	10	9 ^#^
PQRS-G	6	8	2	NS	NS
Crack an egg without shells *					
COPM performance C	7	8	1	NS	NS
COPM satisfaction C	10	9	−1	NS	NS
COPM performance P	6	8	2 ^#^	9	3 ^#^
COPM satisfaction P	8	7	−1	10	2 ^#^
PQRS-G	2	8	6 ^##^	9	7 ^##^
Knit a square *					
COPM performance C	8	7	−1	NS	NS
COPM satisfaction C	7	6	−1	NS	NS
COPM performance P	7	7	0	6	−1
COPM satisfaction P	2	6	4 ^#^	4	2 ^#^
PQRS-G	4	8	4 ^##^	NS	NS

* Practiced goals; ^#^ clinically meaningful COPM improvement in score of 2 or more; ^##^ clinically meaningful PQRS-G improvement in score of 3 or more; C = child-rated; P = caregiver-rated; COPM = Canadian Occupational Performance Measure; PQRS-G = Performance Quality Rating Scale Generic; NS = not scored.

## Data Availability

The datasets in this article are not readily available due to ethical reasons. Requests to access the datasets should be directed to the corresponding author.

## Supplementary Materials

The following supporting information can be downloaded at: https://www.mdpi.com/article/10.3390/children13091257/s1, File S1: Feasibility criteria and data sources; File S2: Interview guides; File S3: Home practice log; File S4: Demographic questionnaire; File S5: Reflexive thematic analysis; File S6: Fidelity scores; File S7: PEM-CY average scores.
