# Supplementary material for: Implementing the Cognitive Orientation to Daily Occupational Performance (CO-OP) Approach with Children with Fetal Alcohol Spectrum Disorder: A Feasibility Study
Source: Children (Basel). 2026 Sep 16;13(9):1257. doi: 10.3390/children13091257 (PMC13605871; doi:10.3390/children13091257)
Supplement: Supplementary file 1 [file children-13-01257-s001.zip › children-4469285-supplementary.pdf]

Supplementary materials

1. Supplement File S1 Feasibility criteria and data sources
2. Supplement File S2 Interview guides
3. Supplement File S3 Home practice log
4. Supplement File S4 Demographic questionnaire
5. Supplement File S5 Reflexive thematic analysis
6. Supplement File S6 Fidelity scores
7. Supplement File S7 PEM-CY average scores

Supplement 1 File S1: Feasibility criteria and data sources

	Feasibility question	Feasibility criterion for success	Data collected
Feasibility of CO-OP	*Acceptability:* Is the CO-OP Approach™ acceptable to participants and caregivers?	80% participants and caregivers report satisfaction with approach<br>80% caregivers report high satisfaction scores on the PAPI	Caregiver semi-structured interviews<br>Child interviews<br>PAPI III
	Practicality (Ease of use): Do child and caregiver participants find CO-OP easy to implement?	80% child and caregiver participants report implementing CO-OP in home practice and sessions easily.<br>50% of participants report at least twice weekly home practice of each goal, on 20 occasions.	Caregiver semi-structured interviews<br>Child interviews<br>Home practice logs
	Implementation: Is the CO-OP implemented per protocol?	>80% Parts A1, A2 and B on CO-OP fidelity checklist of all participants<br>All participants complete outcome measures and other data<br>>80% participants attend screening, outcome measurement appointments and 10 sessions	CO-OP fidelity checklist<br>Completed measures and all protocol data.<br>Video data<br>Field notes and reflective journal<br>Early withdrawal survey

		>80% participants had PRIME-O scores above 30/40	
	Expansion: Can children with FASD use CO-OP to address occupational performance and participation goals?	80% participants identify 3 goals using the COPM and rate these.<br>80% participants make positive changes in at least 1 practiced goal (i.e., PQRS and Performance and Satisfaction scores change >2)	COPM performance and satisfaction scores reported by children and caregivers<br>PQRS-G scores<br>Video data<br>Field notes and reflective journal
	Preliminary efficacy: Does CO-OP improve child and caregiver rated performance and satisfaction scores on identified goals, quality of goal performance participation?	Positive changes are observed in outcome measures (COPM, PQRS-G, PEM-CY) for 80% participants	COPM performance and satisfaction scores reported by children and caregivers<br>PQRS-G scores<br>PEM-CY scores
	Demand: How many participants enrol in this study?	Total number of participants that enrol. Aiming to seek 6-10 participants.	Enrolment records
Feasibility of study design	*Recruitment rate:*	80% participants screened are eligible to participate	Attendance records<br>Recruitment records

	Participation rate: Do all eligible participants who agree to participate complete CO-OP?	80% of participants who agree to participate complete all 10 CO-OP appointments.	Attendance records
	Data collection: Can planned data be collected a baseline (T1), post-training (T2) and 3 months' post training (T3)?	80% all outcome measures collected from all participants	Completed BOT2, VABS3, COPM, PQRS, YC-PEM, PAPI and demographic questionnaires.<br>Field notes

Supplement File S2: Caregiver and children interview guides

Post-CO-OP Therapy Caregiver Interview Guide

Please let the researchers know whether you would like CH or another member of the team to conduct this interview by emailing xx@myacu.edu.au or xx@acu.edu.au

You can choose to have this interview video, audio or note recorded. The recording (video, audio, or notes) will be stored on a password protected database within Australian Catholic University and allocated a code to link this to your other research records. Only the research team will have access to this recording to help us analyse participant experiences of CO-OP. Only themes of the information you provide will be reported and will not identify you or your child. No one else is allowed to use this information. Information will be kept for a minimum of 15 years from the date of publication as required by the Australian Code for the Responsible Conduct of Research.

A separate 1-hour interview will be arranged online via Zoom or Microsoft Teams at a time that is mutually convenient after the project concludes.

The interview will ask questions such as:

1. Tell me about your experience of receiving CO-OP with your child.
2. What was it like for you when helping to support your child use the strategies during the sessions and at other times?
3. How did your child cope with participating in CO-OP?
4. What strategies did your child use that were most helpful to perform goals successfully?
5. How did your child learn to use these strategies?
6. Has your child continued to use the Goal Plan Do Check strategy?
7. What advice would you give to professionals/ researchers for future use of the CO-OP with children with FASD?
8. Would you recommend CO-OP to other families?

Post CO-OP Therapy Interview Guide with Children

These questions will be explored with your child at the end of the last therapy session for 10-15 minutes after they have re-rated their own goals. If you child is unable or unwilling to answer them at this time, we may send a follow up electronic survey that they can choose to complete at another time.

	Did you……	No	A little bit	Yes	A lot	I don't know
1	Like doing CO-OP?	×				?
2	Learn new ways to do things?	×				?
3	Use Goal Plan Do Check and other stategies to do things at home?	×				?
4	Use Goal Plan Do Check and other stategies to do things at school?	×				?
5	Use the Goal Plan Do Check and other stategies to do things in other places (e.g., sport)?	×				?

6. What did you like about CO-OP?
7. What didn't you like about it?
8. Was anything tricky? If yes, what?

	Do you……	No	A little bit	Yes	A lot	I don't know
9	Think CO-OP might help other kids with FASD do activities better?	×				?

10. Why? / Why not?

Your child will be offered a choice to either (i) video, (ii) audio (iii) complete online at home or (iv) have notes recorded without video or audio recording. Interview recording (video, audio, electronic or notes) will be stored on a password protected database within Australian Catholic University and allocated a code to link this to your child's other research records. Only the research team will have access to this recording to help us analyse participant experiences of CO-OP. Only themes of the information your child provides will be reported and will not identify your child. No one else is allowed to use this information. Information will be kept for a minimum of 15 years from the date of publication as required by the Australian Code for the Responsible Conduct of Research.

3-month Follow-Up Interview Guide Caregivers and Children

During the 3-month follow up, we would like to interview your child again with their parent/caregiver, briefly about their continued use of CO-OP and thoughts about it.

They can choose to have this interview video, audio or note recorded. The recording (video, audio, or notes) will be stored on a password protected database within Australian Catholic University and allocated a code to link this to your other research records. Only the research team will have access to this recording to help us analyse participant experiences of CO-OP. Only themes of the information you provide will be reported and will not identify you or your child. No one else is allowed to use this information. Information will be kept for a minimum of 15 years from the date of publication as required by the Australian Code for the Responsible Conduct of Research.

The interview will ask questions such as:

1. Have you continued to use Goal Plan Do Check?
2. Do you think having a CO-OP booster (i.e. more sessions) would be helpful, if so, what frequency and for how long?
3. What/who has helped you use Goal Plan Do Check since we stopped CO-OP sessions?
4. What has been difficult for you to use Goal Plan Do Check since we stopped CO-OP sessions?
5. Have you used Goal Plan Do Check to achieve other goals/tasks?
6. Have you child used Goal Plan Do Check or the previous goals in other environments e.g. at school?
7. What do you think of CO-OP now?

Supplement File S3: Home practice log

This week I have: Date:______

Looked at my goal sheet

- ☐ All the time
- ☐ Sometime
- ☐ Not at all

Tried to use Goal Plan Do Check

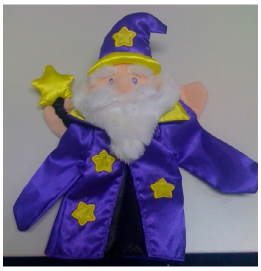

- ☐ All the time
- ☐ Sometime
- ☐ Not at all
- ☐ On my own
- ☐ With help from ______________

How did I go?

____

____

____

Goal 1: ______________________

Did I practice it? YES NO

Where did I do it: ______________________

When did I do it:

- ☐ Everyday
- ☐ When I remember
- ☐ On these days or times: _______________

What plans did I try out?

____

____

How did I go?

____

____

Other information about my goal

____

____

Goal 2: ______________________

Did I practice it? YES NO

Where did I do it: ______________________

When did I do it:

- ☐ Everyday
- ☐ When I remember
- ☐ On these days or times: _______________

What plans did I try out?

____

____

How did I go?

____

____

Other information about my goal

____

____

Goal 3: _______________________

Did I practice it? YES NO

Where did I do it: _______________________

When did I do it:

- ☐ Everyday
- ☐ When I remember
- ☐ On these days or times: _______________

What plans did I try out?

____

____

How did I go?

____

____

Other information about my goal

____

____

Supplement File S4: Child and caregiver demographic questionnaires

Child Demographic Information

Child Background Information

Please complete this background information about [part_b_screening_arm_1][child_fname].

Thank you!

[part_b_screening_arm_1][child_fname]'s gender	○ Male<br>○ Female<br>○ Nonbinary
[part_b_screening_arm_1][child_fname]'s age	____________________
[part_b_screening_arm_1][child_fname]'s grade level at school	____________________
[part_b_screening_arm_1][child_fname]'s living arrangement (please describe e.g., does [part_b_screening_arm_1][child_fname] live in > 1 household?	__________________________
Languages spoken at home (please list any other than English)	____________________
[part_b_screening_arm_1][child_fname]'s age at FASD diagnosis	____________________
Who made the diagnosis	○ Paediatrician<br>○ Child psychiatrist<br>○ Other
Please specify	____________________
What neurodevelopmental domains were impacted (assessed as severe i.e., < 3rd percentile on a standardised test)	☐ Brain structure/neurology<br>☐ Motor skills<br>☐ Cognition<br>☐ Language<br>☐ Academic achievement<br>☐ Memory<br>☐ Attention<br>☐ Executive function<br>☐ Affect regulation<br>☐ Adaptive behaviour, social skills or social communication
Please add details (if known)	__________________________

Primary Carer Demographic Information

Carer’s ID

Carer’s gender Male Female Nonbinary

Carer relationship to child e.g., biological parent, kinship carer, adopted parent, foster carer, other

Family living arrangements who lives with your family and how often?

Name

Relationship to child

Frequency (all the time, weekdays, weekends, school holidays only, fortnightly, other)

Languages spoken at home (other than English)

Employment status

Full-time

Part-time

Studying

Unemployed

Disability pension

Other

Education level

≤ Year 10

Year 12

Diploma

Undergraduate degree

Post graduate degree

Supplement File S5: Thematic Analysis

P1 and C1, P2 and C2, P3 and C3

Inductive, reflexive thematic analysis first then deductive analysis used against Bowen's feasibility criteria (colour coding).

Research Question
What are the perspectives of children (aged 6-12 years) with FASD and caregivers about their participation in the CO-OP intervention?

Theme	Sub-theme	Codes	Examples
**Learning and accepting CO-OP**<br><br>Not like previous therapy, education and family approaches.	Not trusting the CO-OP process (P1 only)<br><br><br><br><br>P3 did trust the process	Learning CO-OP<br><br>Learning CO-OP<br>Learning CO-OP<br><br><br>Learning CO-OP<br><br><br><br><br>CO-OP is different<br>CO-OP is different<br><br>CO-OP is different<br><br><br>CO-OP is different<br>CO-OP is different<br>Parent experience & perspective of CO-OP	What one thing I did sort of find with it was that she needs a little bit more direction (P1).<br>If you don't know what to do, you need a bit more instruction (P1)<br>And so, with this [CO-OP] approach, like with the buttering, I think that, umm, for her to learn one specific way to do it (P1)<br><br>[CO-OP] makes it so that you can sort of order your thoughts for her or for her, because her brain's a bit scattering a lot, like a lot of bubbles all over the place, and she finds it hard to sort of think about what she has to do…and so [the structure] helps her to have an order of how to think (P3)<br><br>She needs more structure and direction in terms of the planning stage. (P1)<br>When the psychologist helped and gave her that thing [reward chart] and was just that extra level of support as well. I think that made a big difference (P1).<br>I know that doing it another way can, we can get their faster. To be shown and told how to do it (P1).<br><br>It's pretty hard to compare it to something else. It was a bit different (P2).<br>[OT] would you recommend it? [P4] Yeah, definitely. Particularly if the traditional methods of OT and things like that don't really work (P3).<br>I guess from me, it was a little bit frustrating with the plan part [of CO-OP] letting her try to do the planning part when it wasn't working… umm so from my perspective it was

			difficult 'cause I could see and it wasn't working…and result in her just getting frustrated and, so um, like a couple of times it was like it's not working so I'm going to show you the steps to do it even though I know that's not technically [CO-OP] (P1).
	Trust and accept CO-OP	CO-OP protocol	I think that any sort of protocol that's stepped out like that is effective to use with her because of what's coming up (P2)<br>it was so targeted, like you have a goal and then you, you plan it, you do it and then you check it (P3).]<br>She knew what, like every time you came, it was a Monday. She says to me, is the egg lady coming today? (P3).<br>you got to tailor your therapy to how the what the child um reacts to positively or the way they the way they will, you know, um, agree to cooperate and engage and everyone's different and you just gotta have that patience and the repeatability of the order that you do things (P3). [CO-OP protocol provides this]<br>You're you're prepared for the types of behaviours you're going to get, and you have strategies to deal with it, and you make it based on what they wanna do more than coming with a kit bag of what you're gonna do (P3). [Protocol facilitated child engagement & goal practice]
		Learning CO-OP	We were doing it more with the hair washing, it was just like wetting the hair, and…. giving her general directions but letting her work it out (P1).
		Learning CO-OP	It's so hard you want them to be, you want her to develop her own plans because she's not going to have somebody standing next to her the whole time through life telling her, giving her specific instructions on how to do everything we want them to develop an ability themselves to develop plans yeah work out how to do things themselves without constantly having to come back to somebody (P1).
		Values CO-OP	Yeah. I will say that the CO-OP approach already sort of aligns, with how [partner] and I sort of see ourselves as parents (P2)

		Accepting CO-OP<br>Accepting CO-OP<br>Accepting CO-OP<br>Accepting CO-OP<br>Accepting CO-OP<br>Accepting CO-OP<br>Accepting CO-OP<br><br>Accepting CO-OP<br><br>Accepting CO-OP	She seemed to enjoy those sessions (P3)<br>I think it was an approach that really worked for her (P3)<br>She really enjoys it and she does it (P3)<br>Did you enjoy doing CO-OP? No (C1) why: Stressful (C1)<br>Did you enjoy doing CO-OP? Yes (C2) I liked learning the approach (C2).<br>Did you enjoy doing CO-OP? A little bit (C3) I liked learning new things (C3)<br>Do you think CO-OP might help other kids with FASD do activities better? Yes. They get to practice doing things (C1).<br>Do you think CO-OP might help other kids with FASD do activities better? A lot. It helps kids get into routine (C2).<br>Do you think CO-OP might help other kids with FASD do activities better? A lot. Helps them learn (C3).
		Child using Goal Plan Do Check	Did you use Goal Plan Do Check to do things at home (OT)? Question mark (C1) I don't remember whether I used GPDC at home, school or other places<br>Did you use Goal Plan Do Check to do things at home (OT)? A little bit (C2)<br>Did you use Goal Plan Do Check to do things at home (OT)? No (C3)
		Caregiver using Goal Plan Do Check	But for me to have a way of getting her to do something, it's good for me to talk to her about something [using Goal Plan Do Check] and then do it in that fashion (P3).
		Use of CO-OP after sessions	I think next year, umm, once she starts school there probably will be some situations where it can probably come in handy. Then particularly the plan and the do part with the stuff at school, what is it that you have to do today, put a plan in place what do you need to do to prepare for today. So, I think in the New Year, so probably more opportunities for her to use the CO-OP method next year in a new environment (P1).<br>Have you used that [CO-O] at all since (OT)? Not really (C2).
		Use of CO-OP after sessions	she doesn't really on her own [goal, plan, do, check]. But, well, if it's something she's interested in, she'll plan and you know, achieve her goals and and check it and stuff (P3).

		Use of CO-OP after sessions	
**Practice influences outcomes**<br>Due to specific neurocognitive differences (memory and learning) more practice is required to support goal achievement	Practice is important<br><br><br><br><br><br><br><br><br><br><br><br><br><br><br><br><br><br>More than 10 CO-OP sessions	CO-OP Practice<br><br>CO-OP Practice<br><br><br>CO-OP Practice<br>CO-OP Practice<br><br><br>Implementing practice<br>Implementing practice<br><br>Implementing practice<br><br><br>More CO-OP<br>More CO-OP<br><br><br>More CO-OP<br><br>More CO-OP<br><br><br><br><br><br>More CO-OP – slow progress	But for the most part, letting her develop how she you know, the plan and everything (P1)<br>I think just, ah, reinforcing of the goals… without the reinforcement it just goes away. It doesn't have to be reinforced a hundred times, just once or twice. Just to put it to the forefront of her mind (P2)<br>the just the repetition of it, like doing the same thing every session (P3).<br>but you're gonna get better at it just from doing it and then like as you get better, you get more confidence (P3).<br><br>But again it's that, you know having time and effort to get to a point where she's better at developing her own plan (P1)<br>Taking turns at cards...I think if that was something we were doing all the time It wouldn't have been an issue, but that, ah, muscle memory of it, I guess, needs a bit of constant reinforcement (P2)<br>[Practice] we did that. We did that more in the knitting as a bedtime activity cause it's so it's kind of you fall asleep while you're doing it quite easily. So it was a good calming sort of activity to do together (P3).<br><br>I think more sessions would probably…we would have got a little bit further (P1).<br>Obviously, you know, people like [C2] it's an ongoing thing. I don't think there could ever be enough sessions. I could have you come here every week for the rest of our lives (P2).<br>I think with what we were doing maybe if it had of been, even half that again so 15 sessions so that would have embedded the skills a bit more (P1).<br>she misses it now that's stopped. Yeah, she could have, like, she could have gone on with, you know, doing it more, a few more, and she might have then conquered a bit more of the knitting, but just having that (P3).<br>That only happened. They only happened towards the end [bike riding]. So if we had all the sessions again, just imagine, she'd be totally in (P3).

	Outcomes of CO-OP	More CO-OP – slow progress<br><br>Outcome – goal achievement<br>Outcome – application of CO-OP<br>Outcome - Creating opportunity<br><br>Outcome - Creating opportunity<br><br>Outcome - Creating opportunity<br><br>Outcome - emotional<br><br>Outcome – small<br><br>Outcome - limited<br><br>Outcome – emotional<br><br>Outcome - emotional<br><br>Outcome - emotional	Like if she saw more progress a little bit quicker, it would have turned a bit more. Yeah, but the knitting's really slow, so it's well for a beginner as you don't see fast enough progress and I guess it's a bit off putting for her (P3).<br>It's a good approach and it makes sense, but I think it makes slow ground (P2).<br><br>We're still working on her goals that we had here [at 6 months follow up] (P1)<br><br>I just said quick, you did it the day before and, she did, she used her shirt to grab it and take it out. It wasn't a big deal, but it's a way that she worked out how to do it. So, she put a plan, she thought of something.. She put a plan into action and she did it....Whereas in the past she would not have done that (P1).<br><br>But because she has been doing it, she has been doing it regularly for now how many weeks has it been now, she's not, she just opens the fridge now (P1).<br>But that's because she didn't have the, she's never experienced a lot of bike riding. She's never been beyond trailer wheels. And all of a sudden you say, rip off the trainer wheels, let's start riding the bike (P3).<br>now she can do everything that you were asking her to try and learn to do. She's really developed a lot (P3).<br><br>So I'm sure that the less she gets reminded about stuff, the more her confidence lifts a bit (P2).<br>In terms of like outcomes, ah, we've come to sort of realise that it's small changes that are big things (P2).<br>She may know how to do something in very specific circumstances but can't apply it to a new situation (P1).<br>She just got it [bike riding without training wheels] all of a sudden, she got inspired and because she started to see that she was making progress, like turning corners without a problem (P3).<br>And yeah, and all of a sudden, she's realising, wow, this is something I really enjoy doing [bike riding] (P3).

		Outcome - emotional<br><br>Outcome – application CO-OP<br>Outcome – goal achievement	just then one day she started to be able to turn corners and like, you know how she was trying to turn that circle. Yeah. And then all of a sudden that worked and then she did a bit more and she went a little bit faster and she realised, gee, this feels really good on my body and yeah, and she really enjoys like getting her little legs and going and she goes racing down the street 100 miles an hour and you can see that she's just loves that adrenaline rush she gets (P3).<br>She got a bit annoyed with that one in the end [knitting], I think because we kept on making her do it and then, like, she didn't really achieve a lot from the amount of time that she was doing it (P3).<br><br>And also when I wanted, I wanted a different colour [knitting]. It was her idea to tie the thing onto the next wool (P3)<br>yeah, I think she's achieved all the goals for. Yeah, definitely. Bike riding. Definitely. That's huge (P3).
**Child engagement in CO-OP**	Learning style and memory impacts application of CO-OP	Impacts on CO-OP<br><br>Impacts on CO-OP<br><br>Impacts on CO-OP<br><br>Impacts on CO-OP<br><br>Impacts on CO-OP<br><br>Impacts on CO-OP<br><br>Impacts on CO-OP	It's a pretty common FASD thing…to analyse and use other information in new situations is a really big issue with her…so I think maybe with practice (P1)<br>I guess with the CO-OP strategy it's, you know there's going to be hurdles, so it's a matter of how you get over those hurdles together, and I think your understanding of how uncooperative it may seem these kids are (P3).<br>Why do you think (you didn't use it)? (OT) I got distracted (C2)<br><br>I keep forgetting [to use GPDC] (C1)<br><br>I also, forgot about that as well [re: checking] (C1)<br><br>Like everything else, it is dependent on a bunch of variables that neither you nor I really know [C2's response to participating in CO-OP]. I think it just came down to the mood on the day (P2).<br><br>[CO-OP] makes it so that you can sort of order your thoughts for her or for her, because her brain's a bit scattering a lot, like a lot of bubbles all over the place, and she finds it hard

			to sort of think about what she has to do…and so [the structure] helps her to have an order of how to think (P3)
	Implementing CO-OP	Impacts on CO-OP	Why do you think [you didn’t use it]? I got distracted (C2)
	Implementing CO-OP	Implementing	I think it, it just took her, it took a bit of time to get her to cooperate, and it sometimes felt that she wasn't really going to do anything (P3).
		Implementing	If the planning isn’t working, she doesn’t know how to modify it (P1)
		Implementing	So you sort of have to do something sensory first [before knitting], like the egg or the bike or this hammock or something. And then and then she might like, agree to do some knitting, but just that repetition (P3).
		Implementing	all she needed was to accept guidance (P3).
	Emotional responses to CO-OP	Decision making is stressful.	Stressful doing [CO-OP: decisions and analysing] (P1)<br>I found CO-OP stressful (C1)
		Enjoyed it<br>Enjoyed it	I liked learning to use CO-OP. There was nothing I didn’t like (C2).<br>she loves the feeling of, of going fast [bike riding without training wheels]. It makes her feel. It makes her feel like she's exerting energy and she needs to get her energy out. And that's a really good way of doing it. (P3)
		Frustration limits tolerance	I don’t really know because she was doing really well for, and then she just had one morning where it was just not happening for her. And from then on, she seems to, and as I said umm on Tuesday, if there was only one shoe. I think she would be better but it’s that frustration level that she doesn't have a huge tolerance for getting frus, like she gets frustrated quite easily (P1)
		Frustration limits tolerance	Or more frustration like the knitting…Yeah, the knitting is, um, I think the fact that she needed so much direction was a little bit of a turn off for her, but she couldn't. She couldn't just pick it up and give it a go and something would happen. You know, actually need help to get started, you need. And then when things go wrong, you, you really need to have that corrected, whereas she was just going on continuing to do it wrong because she couldn't be

	Stamina and fatigue limits participation	Frustration leads to self-harm<br><br>Cognitive & physical fatigue<br><br>Cognitive & physical fatigue<br><br>Cognitive & physical fatigue<br><br>Cognitive & physical fatigue	bothered figuring it out because that would mean asking someone or, you know, focusing (P3).<br><br>That frustration builds to the point where she starts screaming or making noise, stomping her feet or hitting herself (P1)<br><br>I didn't realise that for kids with FASD stamina and physical fatigue is something that's an issue….And the fact by afternoon she's tired, she's spent all day you know, trying to keep it together, trying to concentrate, trying to follow instructions, trying to make sure that she's got what she needs, where she needs to be. So, by the afternoon she's tired (P1).<br><br>She'll run out of oomph! Because that's the other thing to, is that she with her in particular, when we're learning new skills, is that she tires quite easily (P1).<br><br>I think that affects her ability to, that then links in. The physical being physical tired links in with her frustration tolerance and her ability to plan and do tasks (P1).<br><br>If she is tired that makes everything more difficult when everything's difficult already (P1).
**Family involvement in CO-OP**	Limited involvement	Limited involvement<br>Limited involvement<br><br>Limited involvement<br><br>Limited involvement	I haven't really used it to be honest with you (P2).<br>Not the best, I'd say. It was more of a consequence of a bit of a hectic, ah, lifestyle we've been living at the moment (P2).<br>With, ah, me working away a lot and [partner] effectively being a single mum with three kids for a lot of the year. There is just not a lot of time to reinforce it and keep going over and over it again (P2).<br><br>Did anyone help you use CO-OP (OT)? No (C2)

	Need for external support<br><br><br><br><br><br><br><br><br><br>Family involvement	Need external support<br><br><br><br>Need external support<br>Need external support<br><br>Parent involvement is important<br><br><br><br><br><br><br><br><br>Cousin involvement	I think we got to a stage where were at where we need someone to do it with us. We've been doing a lot over the years. A lot of stuff just the two of us…to the point now where she is, she finds it frustrating and gets angry with me. If [OT] had come over a few more times you would have got a little bit better at it than you are because you don't do it anytime that I ask you to do it [talking to C1] (P1)<br>It's just getting someone else in to work on a specific skill (P1)<br><br>You need that extra support, having somebody else come in and help support certain skills (P1)<br><br>And that they really need the parents to participate there as well, because otherwise they just take over, do their own thing more, whereas I, I can keep her down, attending to it, but not whether or not it's a, you know, it's worth it. You don't always get the same outcomes from it, but you're more likely to get them to attend if if you're there with them. Yeah, that's so so important to because then the therapist teaches you how to continue to do it in because they're not there every day and they're only there for a short period of time. So to have that knowledge and um, skill and to see that it works. Um, it's really, it's really helpful for. It was really helpful for me anyway.<br><br>And the other thing I think that was helpful was when [cousin] said to her, um, uh, she has no clue on how to ride a bike and that she wanted you to give her lessons. I think that was a clincher because [cousin]'s better at [C3] in virtually everything. And all of a sudden, [C3]'s like, I can ride a bike and [cousin]'s going, I wish I could be like you. And that was really, really critical (P3).
**Goals**	Choice motivates		I think its [choosing her own goals] good because, umm, they're things that she finds frustrating. Her being able to choose things and then she is more likely to practice it and it's gonna have a bigger impact on her life because they are things that frustrate her (P1).<br>Especially when I can see goals that she's set for herself because she obviously has her own agenda to achieve them (P2)<br>That [handwriting goal] has been a bit of a stayer, there hasn't been much prompting involved in that (P2)

			You'd think that she'd be much more inclined to engage with it if it's on her terms, which we know that's how she operates. Everything has to be on her terms (P3).<br>It always went back to her chosen one was what she wanted to do (P3).<br>I think that's very important because if you want to get something out of the session in terms of her engagement and her motivation, then it has to be, it has to be from what she wants to do (P3).
	Number of goals		And there's so much going on, as I say, that the other thing that, she' really been working on, well there are the three skills (P1)<br>Really, it's a lot of skills to work on in 10 sessions (P1)<br>Maybe less, focussing on 1 or 2 things rather than doing more (P1)<br>Because we have been so focused on that [three goals for CO-OP] that we haven't really looked at trying to learn any new skills (P1)<br>Too much [too many goals] (C1)
	Everyday life		And obviously that goal [taking turns in UNO] is going to be less of a priority….It's not going to be the number one thing. Whereas with the handwriting, um, because its, she writes everyday. It's not an issue (P2).<br>Picking stuff [i.e. goals] that comes up a bit (P2)<br>I'd say, um, yeah like that, that's probably an approach that [partner] probably uses without realising it. So that, I mean that happens on a weekly basis with cleaning your room (P2)
**Working towards child independence and participation**			She is more independent now. She only needs little reminders (P2).<br>So having a strategy like that I think is helpful, even though it doesn't show like you don't realise it at the time. I think if you can, if you continue to do it in that way, then it builds up her sort of ability to do it herself (P3)<br>And so now she's working on trying to get up hills without stopping (P3).<br>And we and we've got the support workers on it as well and they all go to the park, and she rides a bike. They go, the support worker goes on [brother]'s scooter, [brother] takes the other scooter and they just all go together, so it's really nice (P3).<br>so she just scoots around a a few blocks and yeah, she's done it in about 20 minutes (P3).<br>When we're playing UNO, she's sort of a bit better at keeping up with the game (P2).

Acceptability – how the intended individual recipients react to the intervention

Implementation – the extent, likelihood and manner in which an intervention can be fully implemented as planned and proposed

Practicality – the extent to which an intervention can be delivered with resources, time, commitment

Expansion – the potential success of an already-successful intervention with a different population

Limited efficacy/perceived outcomes – immediate rather than final outcomes, with shorter follow up.

Supplement File S6: Fidelity scores for each participant across sessions

Josie	Total	Percentage	S1 total	S1 percentage	S6 total	S6 percentage	S8 total	S8 percentage
A1: Across sessions	24	80						
A2: Within session			28	80	45	82	44	80
General fidelity			32	91	24	96	27	90
Olivia	Total	Percentage	S1 total	S1 percentage	S6 total	S6 percentage	S8 total	S8 percentage
A1: Across sessions	24	80						
A2: Within session			46	84	46	84	42	76
General fidelity			37	93	34	97	28	93
Sarah	Total	Percentage	S1 total	S1 percentage	S6 total	S6 percentage	S8 total	S8 percentage
A1: Across sessions	14	93						
A2: Within session			31	78	46	84	40	80
General fidelity			27	78	34	97	34	97

S1 = session 1

S6 = session 6

S8 = session 8

Supplement File S7: Average frequency of participation and level of involvement scores overtime rated on the PEM-CY

	Josie			Olivia			Sarah		
	T1	T2	T3	T1	T2	T3	T1	T2	T3
Home participation*	5.8	6.5	6.5	6.1	5.8	4.4	5.1	6.2	5.3
School participation*	5.3	4.8	5.3	4.3	4.6	2.8	3.5	3.5	2.8
Community participation*	5.2	4.7	5.2	4.2	NS	3.8	3.2	4	2.7
Home**	4	4.4	4.3	3	3.6	3.1	2.9	3.6	3.7
School**	3	4	3.7	2.3	NS	3	1.2	2.3	2.4
Community**	4.5	5	4	3	NS	4	4.2	2.5	2.1

*average frequency of participation score (0= never, 7 = daily)

** average involvement score (1 = minimally involved, 5 = very involved)

NS = no score
